# Immunomodulatory Nanoparticles Induce Autophagy in Macrophages and Reduce *Mycobacterium tuberculosis* Burden in the Lungs of Mice

**DOI:** 10.1021/acsinfecdis.4c00713

**Published:** 2025-02-25

**Authors:** Raymonde B. Bekale, Retsepile E. Maphasa, Sarah D’Souza, Nai Jen Hsu, Avril Walters, Naomi Okugbeni, Craig Kinnear, Muazzam Jacobs, Samantha L. Sampson, Mervin Meyer, Gene D. Morse, Admire Dube

**Affiliations:** † Infectious Disease Nanomedicine Research Group, School of Pharmacy, University of the Western Cape, Cape Town 7535, South Africa; ‡ Division of Immunology, Department of Pathology, University of Cape Town, Cape Town 7701, South Africa; § National Health Laboratory Service, Cape Town 8005, South Africa; ∥ Neuroscience Institute, University of Cape Town, Observatory 7925, South Africa; ⊥ Institute of Infectious Disease and Molecular Medicine, Faculty of Health Sciences, University of Cape Town, Observatory, Cape Town 7925, South Africa; # DSI-NRF Centre of Excellence for Biomedical Tuberculosis Research, South African Medical Research Council Centre for Tuberculosis Research, Division of Molecular Biology and Human Genetics, Faculty of Medicine and Health Sciences, Stellenbosch University, Cape Town 7505, South Africa; ∇ South African Medical Research Council Genomics Platform, Tygerberg, Cape Town 7501, South Africa; ○ Department of Science and Innovation/Mintek Nanotechnology Innovation Centre, Biolabels Node, Department of Biotechnology, University of the Western Cape, Cape Town 7535, South Africa; ◆ Center for Integrated Global Biomedical Sciences, School of Pharmacy and Pharmaceutical Sciences, University at Buffalo, State University of New York, Buffalo, New York 14215, United States

**Keywords:** Mycobacterium tuberculosis, immunotherapy, host-directed therapy, autophagy induction, innate immunity, PLGA nanoparticles, immune modulation, Curdlan-PLGA nanoparticles, tuberculosis treatment

## Abstract

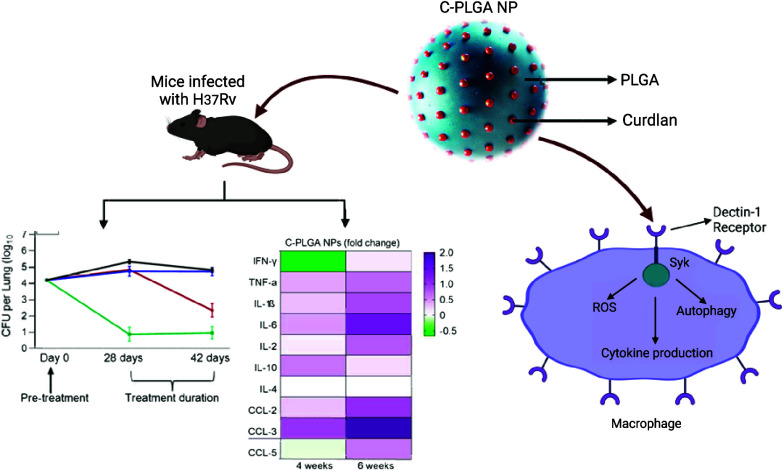

Tuberculosis (TB) is the leading cause of death from infectious disease. Macrophages are the primary immune responders and become the primary host cells for the causative agent *Mycobacterium tuberculosis*. Following the uptake of *M. tuberculosis*, the inherent antimicrobial action of macrophages is dampened, enabling the bacterium to reside within these cells and multiply. Rising resistance of *M. tuberculosis* to antibiotics has led to the investigation of novel approaches for the treatment of TB. Here, we report a host-directed approach, employing biomimetic Curdlan poly(lactic-*co*-glycolic acid) (C-PLGA) nanoparticles (NPs), and examine autophagy induction in infected macrophages, eradication of *M. tuberculosis* and immune modulation in a mouse model. We demonstrate that the NPs induce autophagy in *M. tuberculosis*-infected macrophages. Treatment of H37Rv infected C57BL/6 mice with these NPs reduced *M. tuberculosis* burden in the lungs of mice and modulated cytokines and chemokines and this work demonstrates that these immunomodulatory NPs are a potential treatment approach for TB.

The number of people dying due to tuberculosis (TB) each year remains unacceptably high. In 2022, about 1.3 million deaths globally were due to TB.^1^ Recent progress made in reducing the number of deaths due to TB has been reversed as a consequence of disruptions to TB diagnosis and treatment during the COVID-19 pandemic. In addition, there is an increase in cases of TB, which cannot be treated using available antibiotics, and there is a relatively small pipeline of new antibiotics under development.^2,3^ Drug resistance can arise soon after the introduction of the drugs into treatment regimens, as was seen with delamanid and bedaquiline.^4^ Therefore, there is a need for therapies with new modes of action, which can potentially bypass microbial resistance.

*Mycobacterium tuberculosis* is predominantly an intracellular microbe and the macrophage is the primary host cell.^5^*M. tuberculosis* suppresses the antimicrobial response of the macrophage through mechanisms, which include limiting phagosome maturation and modulating cytokine and reactive oxygen and nitrogen species (ROS/RNS) production, and suppressing autophagy.^5−7^ This suppression of the macrophage defense response ensures the survival and replication of the pathogen within the cells. Therefore, there is an opportunity to tilt the scales in favor of the macrophage by activating macrophages into an antibacterial state.^8^ Host-directed therapies have been proposed as potential novel treatments for TB^9^ and a number of compounds such as vitamin D3 and repurposed drugs such as atorvastatin are under clinical investigation as adjunctive therapies in combination with standard anti-TB drugs.^10^

To add to these approaches and to investigate *M. tuberculosis* eradication in the absence of antibiotics, we have developed a poly(lactic-*co*-glycolic acid) (PLGA) nanoparticle (NP) surface-functionalized with Curdlan (a 1,3- β-glucan) derived from *Alcaligenes faecalis*, as a host-directed immunotherapeutic treatment for TB. The presence of Curdlan enables greater NP interaction with the pattern recognition receptor Dectin-1 found on macrophage and dendritic cell surfaces.^11^ Binding of Curdlan to Dectin-1 is known to activate various downstream signal transduction pathways that promote proinflammatory gene expression as well as intracellular reactive oxygen and nitrogen species (ROS/RNS) production. Proinflammatory cytokines produced through Dectin-1 activation include IL-12 and TNF-α,^12,13^ which are crucial to the control of TB.^14,15^ We have previously reported on the synthesis of C-PLGA NPs and that these NPs are noncytotoxic and modulate cytokines and chemokines in macrophages and lead to a reduction in *M. tuberculosis* burden in macrophages.^16−18^ From the *in vitro* data, confirmation in an immune environment is needed and requires a sentient animal model with immune and physiological responses. Mice, which are well-established models in immunology and toxicology, were selected to enable a more comprehensive understanding of the effects of these NPs.

Here, for the first time, we report the treatment effect of Curdlan in an animal model of *M. tuberculosis* infection. Curdlan is presented in a particulate form on the PLGA NP surfaces. We hypothesized that Curdlan functionalized PLGA NPs can stimulate immune cells, induce autophagy in macrophages, increase intracellular production of cytokines, and eliminate *M. tuberculosis* in infected mice.

## Results and Discussion

### Nanoparticle Synthesis

**Figure 1:**
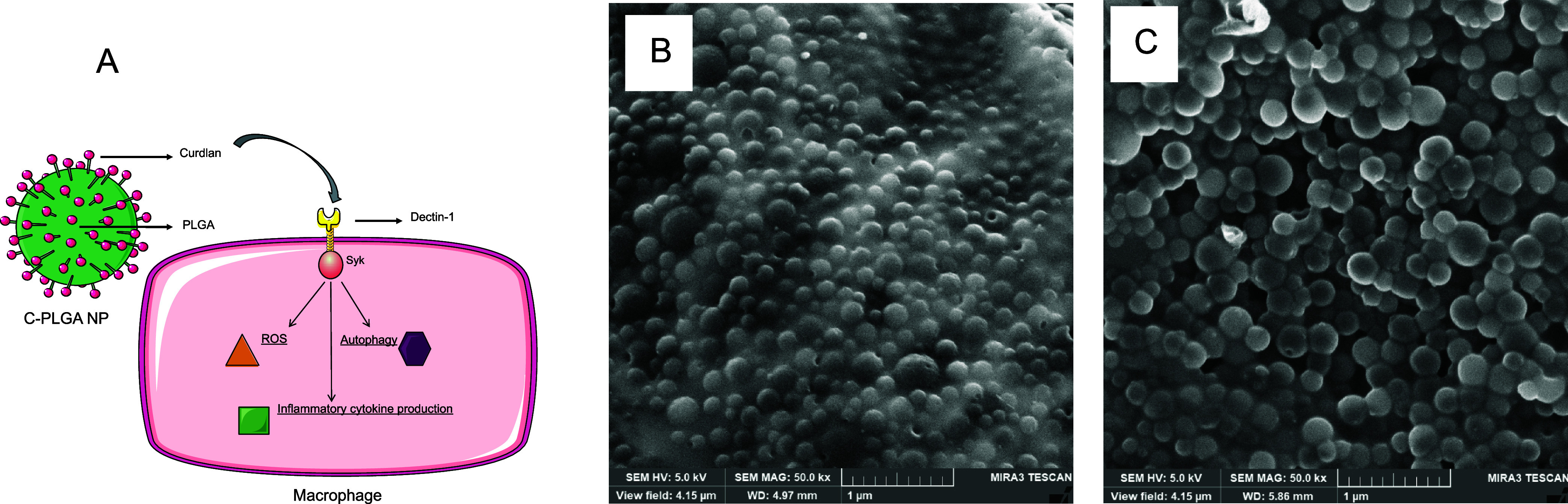
Schematic representation of the approach of C-PLGA NPs as systems to activate macrophages primarily through the Dectin-1 receptor (A). SEM images show the morphologies of PLGA NPs (B) and C-PLGA NPs (C).

In this study, two types of NPs were synthesized, i.e., PLGA and C-PLGA (functionalized with 8% w/w Curdlan). In prior work,^16^ we have shown that in C-PLGA NPs, Curdlan is on the surface of the NPs and can interact with macrophage receptors including Dectin-1 (Figure 1A). We characterized the properties of the NPs in water, D10 (i.e., complete DMEM medium supplemented with 10% fetal bovine serum (FBS) and 1% Penicillin–Streptomycin which was used to suspend particles for *in vitro* studies), and saline (media used to suspend particles for animal studies). PLGA NPs showed hydrodynamic diameters (Hd) ranging from 253 ± 7.5 to 377.7 ± 30.33 nm in the various media, while C-PLGA NPs showed Hd ranging from 303.9 ± 5.3 to 463.9 ± 22.61 nm (Table 1). As expected, a spherical shape of the NPs was observed (Figure 1B,C), and core particle diameters as measured by scanning electron microscopy (SEM) were smaller than the Hd (202.9 ± 29.6 nm and 267.8 ± 38.9 for PLGA NPs and C-PLGA NPs, respectively). The overlap in size distribution and ζ-potential profiles between the PLGA and C-PLGA NPs indicated that particle properties were similar across the particle types. For macrophage uptake studies, NPs were loaded with the hydrophobic stain 3,3′-dioctadecyloxacarbocyanine perchlorate (DiO) and properties were 356.3 ± 5.2 nm (PDI = 0.20 ± 0.10; ζ-potential = −15.2 ± 0.51 mV) and 403.5 ± 40.1 nm (PDI = 0.35 ± 0.05; ζ-potential = −11.8 ± 0.36 mV), for PLGA and C-PLGA NPs, respectively.

**Table 1:** Physicochemical Properties of PLGA NPs and C-PLGA NPs Synthesized Using a Single Emulsion–Solvent Evaporation Method as Measured in Water, DMEM, and Normal Saline^a^

particle type	Hd (nm)	PDI	ζ-potential (mV)
PLGA NPs	deionized water		
	346.8 ± 9.81	0.24 ± 0.03	–20.93 ± 4.03
C-PLGA NPs	371.8 ± 25.28	0.36 ± 0.04	–16.95 ± 8.42
	D10		
PLGA NPs	253 ± 7.5	0.39 ± 0.01	–7.29 ± 0.76
C-PLGA NPs	303.9 ± 5.3	0.34 ± 0.05	–6.34 ± 0.25
	saline		
PLGA NPs	377.7 ± 30.33	0.19 ± 0.01	–18.63 ± 0.99
C-PLGA NPs	463.9 ± 22.61	0.31 ± 0.07	–13.90 ± 4.42

a Results shown are mean ± standard deviation (SD) of 3 replicates.

### Nanoparticles Induce Autophagy in *M. tuberculosis*-Infected Macrophages

**Figure 2:**
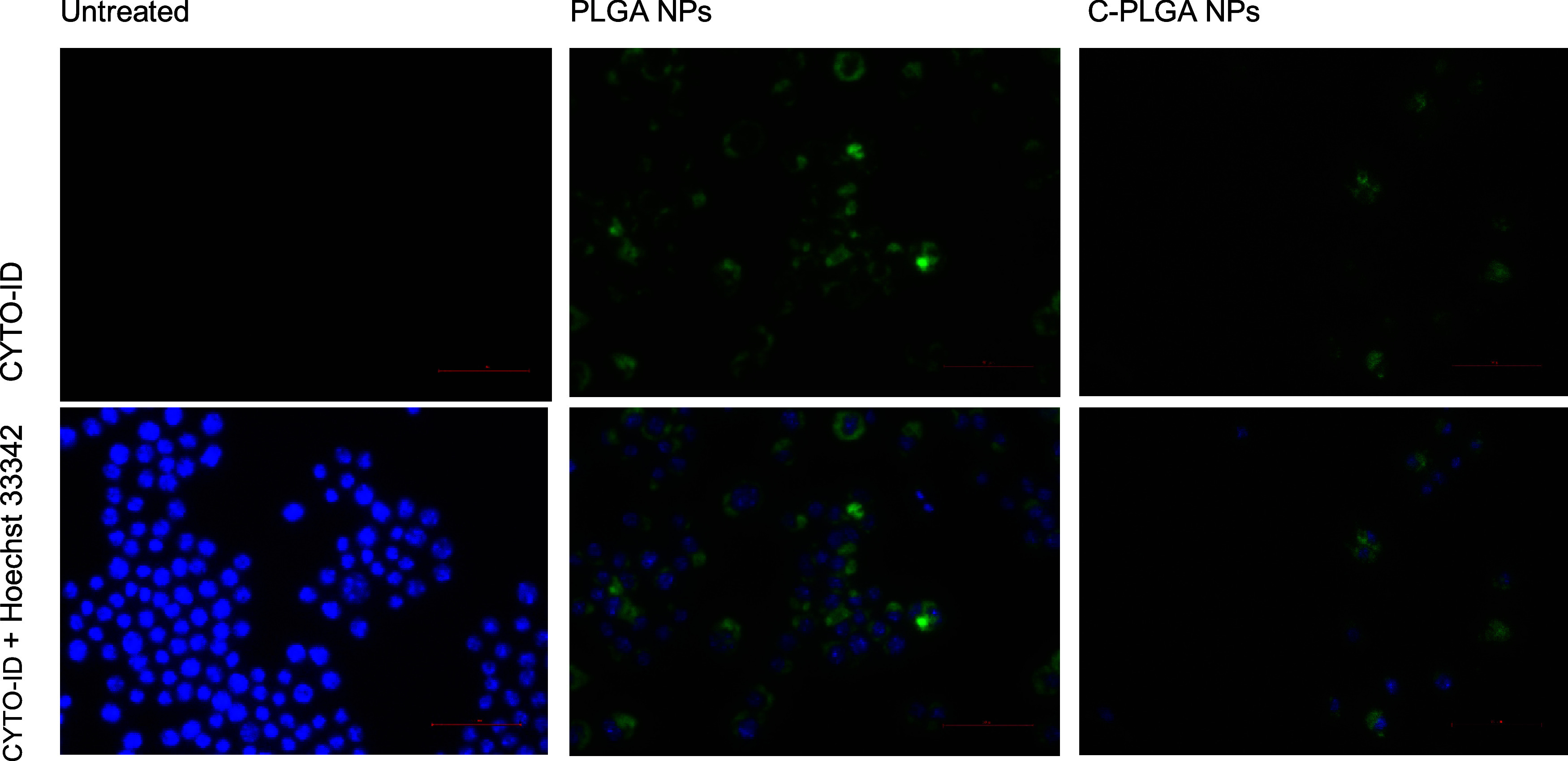
Representative fluorescence microscopy images of RAW 264.7 macrophages stained with the autophagic CYTO-ID Green dye and double stained with the CYTO-ID Green dye and the Hoechst 33342 nuclear stain, after a 24 h treatment period with 1 mg/mL of PLGA NPs and C-PLGA NPs. Scale bar: 50 μm.

**Figure 3:**
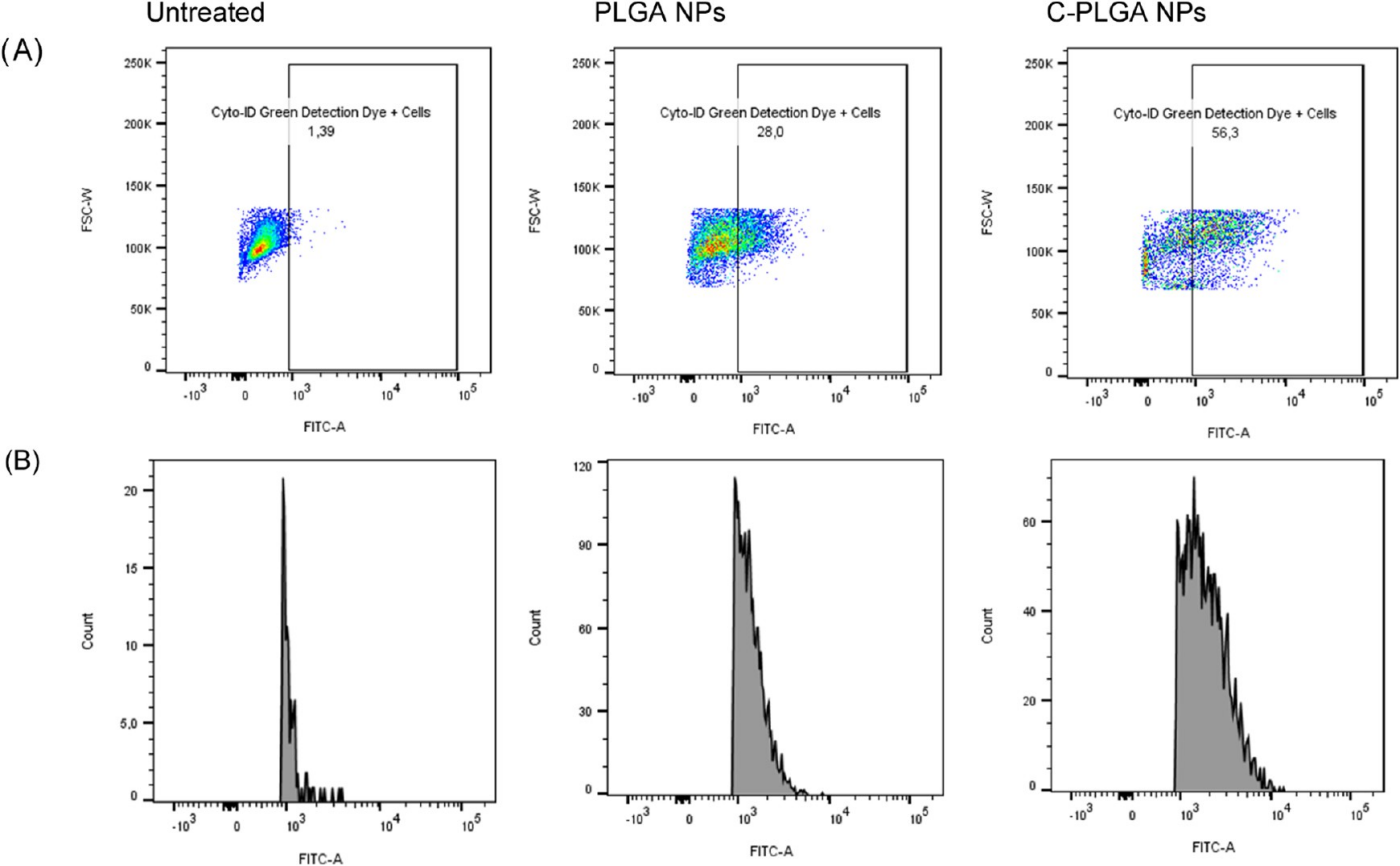
Flow cytometry data presented as (A) Fluorescein isothiocyanate (FITC) scatter plots and (B) FITC histograms to compare the amount of induced autophagy after 24 h of treatment with PLGA and C-PLGA NPs, compared to untreated macrophages. Each treatment has three replicates and the results with the highest values were chosen to represent the replicates, *n* = 3.

We first sought to investigate whether the NPs could induce autophagy in *M. tuberculosis*-infected macrophages, which could be linked as a possible mechanism for the eradication of the bacterium. In uninfected macrophages, autophagy vesicles (fluorescently labeled with CYTO-ID) were found to accumulate as both green foci distributed throughout the cytoplasm and as green spherical vacuoles on the perinuclear regions of PLGA NPs and C-PLGA NP-treated macrophages. Autophagy was not present in the control macrophages (Figure 2). Flow cytometry data showed negligible fluorescence of the CYTO-ID green dye in untreated macrophages while showing significant fluorescence in both PLGA NP and C-PLGA NP-treated macrophages (Figure 3). The untreated macrophages were gated to only allow for background fluorescence of 1.15 ± 0.21%, to represent any naturally occurring autophagy for housekeeping purposes in the macrophages. When the same gating strategy was applied to macrophages exposed to the NPs, an increase in the percentage of macrophages stained with CYTO-ID green dye to 23.57 ± 3.87% and 53.30 ± 3.82% was observed for the macrophages treated with PLGA NPs and C-PLGA NPs, respectively.

**Figure 4:**
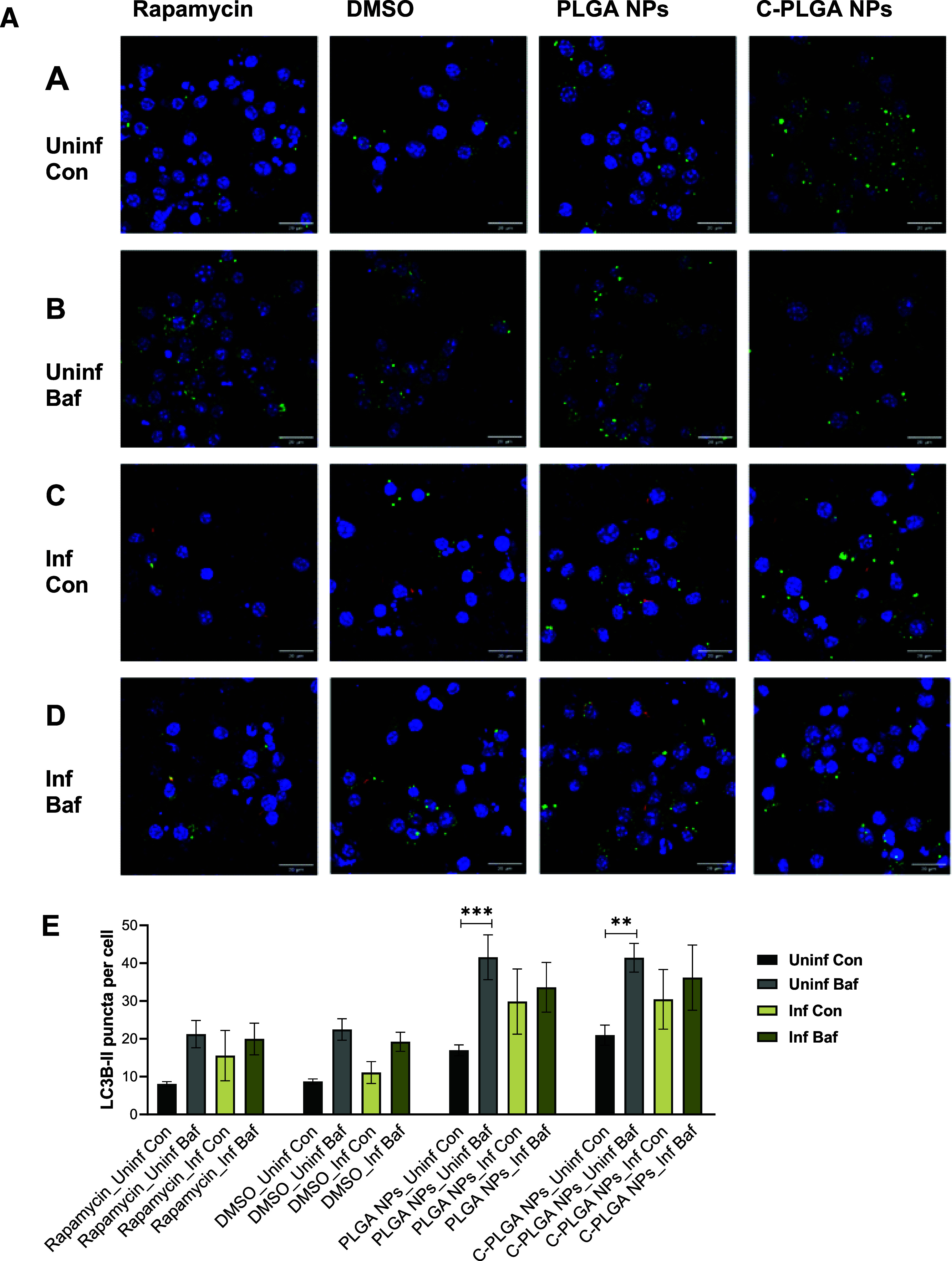

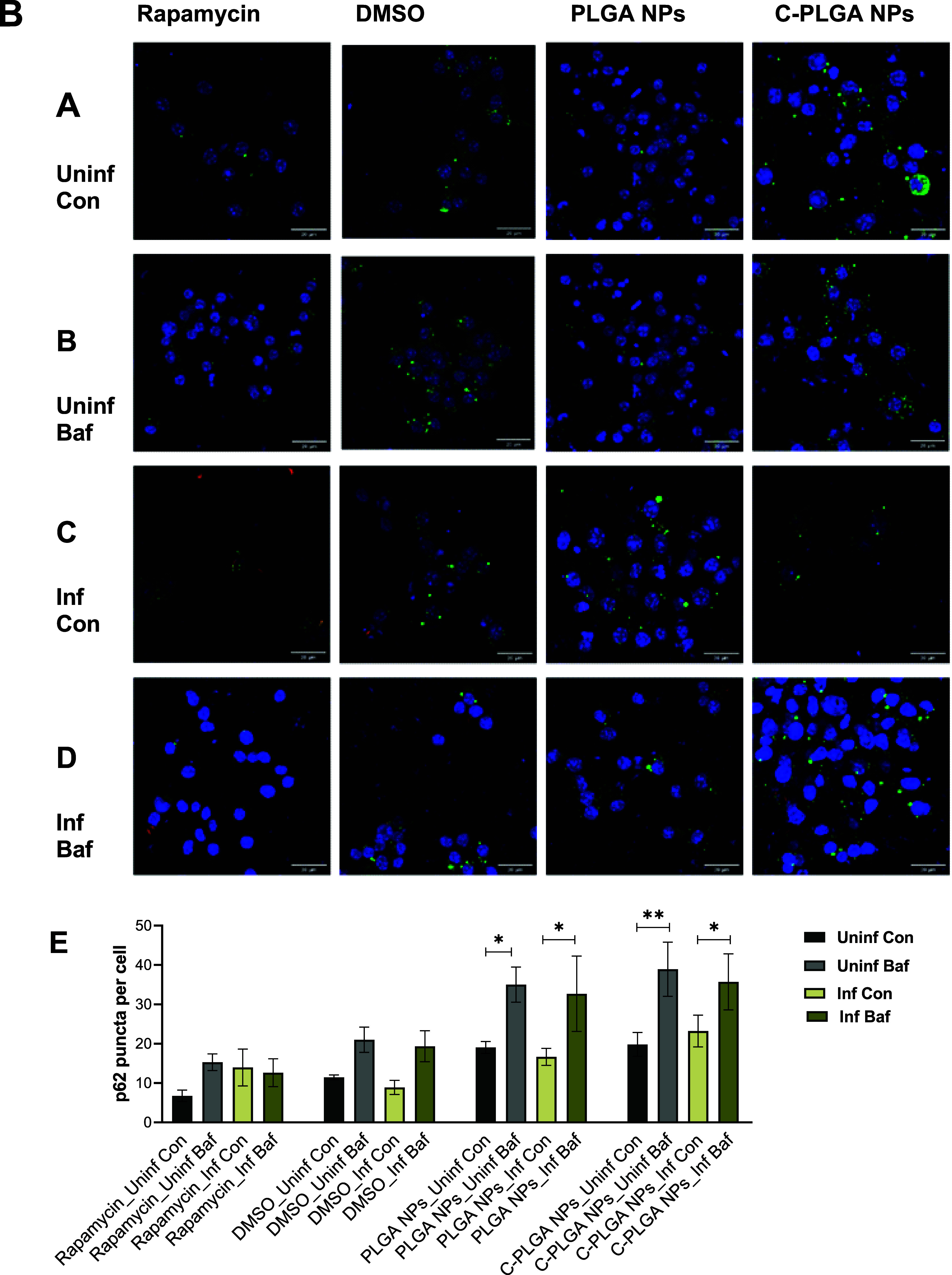
(A) Confocal microscopy images showing the punctate green structures of the LC3B-II autophagy marker in uninfected (A and B) and *M. tuberculosis*-infected (C and D) RAW 264.7 macrophages after a 24 h treatment with Rapamycin, DMSO, PLGA NPs, and C-PLGA NPs, with or without Bafilomycin. The intracellular LC3B-II appear in the images as green spherical autophagy vesicles (punctate structures). The punctate structures are localized around the blue nuclei, as stained by the Hoechst nuclear stain. In *M. tuberculosis*-infected cells, red/pink-like structures (pointed by white arrows) indicate the presence of the bacteria. Quantitative analysis of LC3B-II puncta per cell (E) in uninfected and *M. tuberculosis*-infected RAW 264.7 macrophages after treatment with Rapamycin, DMSO, PLGA NPs, and C-PLGA NPs, with or without Bafilomycin. Scale bar = 20 μm. *n* = 3 for each condition. (*) = *P* ≤ 0.05, (**) = *P* ≤ 0.01, and (***) = *P* ≤ 0.001. (B) Confocal microscopy images showing the punctate green structures of the p62 autophagy marker in uninfected (A and B) and *M. tuberculosis*-infected (C and D) RAW 264.7 macrophages after a 24 h treatment with Rapamycin, DMSO, PLGA NPs, and C-PLGA NPs, with or without Bafilomycin. The intracellular p62 appears in the images as green spherical autophagy vesicles (punctate structures). The punctate structures are localized around the blue nuclei, as stained by the Hoechst nuclear stain. In *M. tuberculosis*-infected cells, red/pink-like structures (pointed by white arrows) indicate the presence of the bacteria. Quantitative analysis of p62 puncta per cell (E) in uninfected and *M. tuberculosis*-infected RAW 264.7 macrophages after treatment with Rapamycin, DMSO, PLGA NPs, and C-PLGA NPs, with or without Bafilomycin. Scale bar = 20 μm. *n* = 3 for each condition. (*) = *P* ≤ 0.05 and (**) = *P* ≤ 0.01.

**Figure 5:**
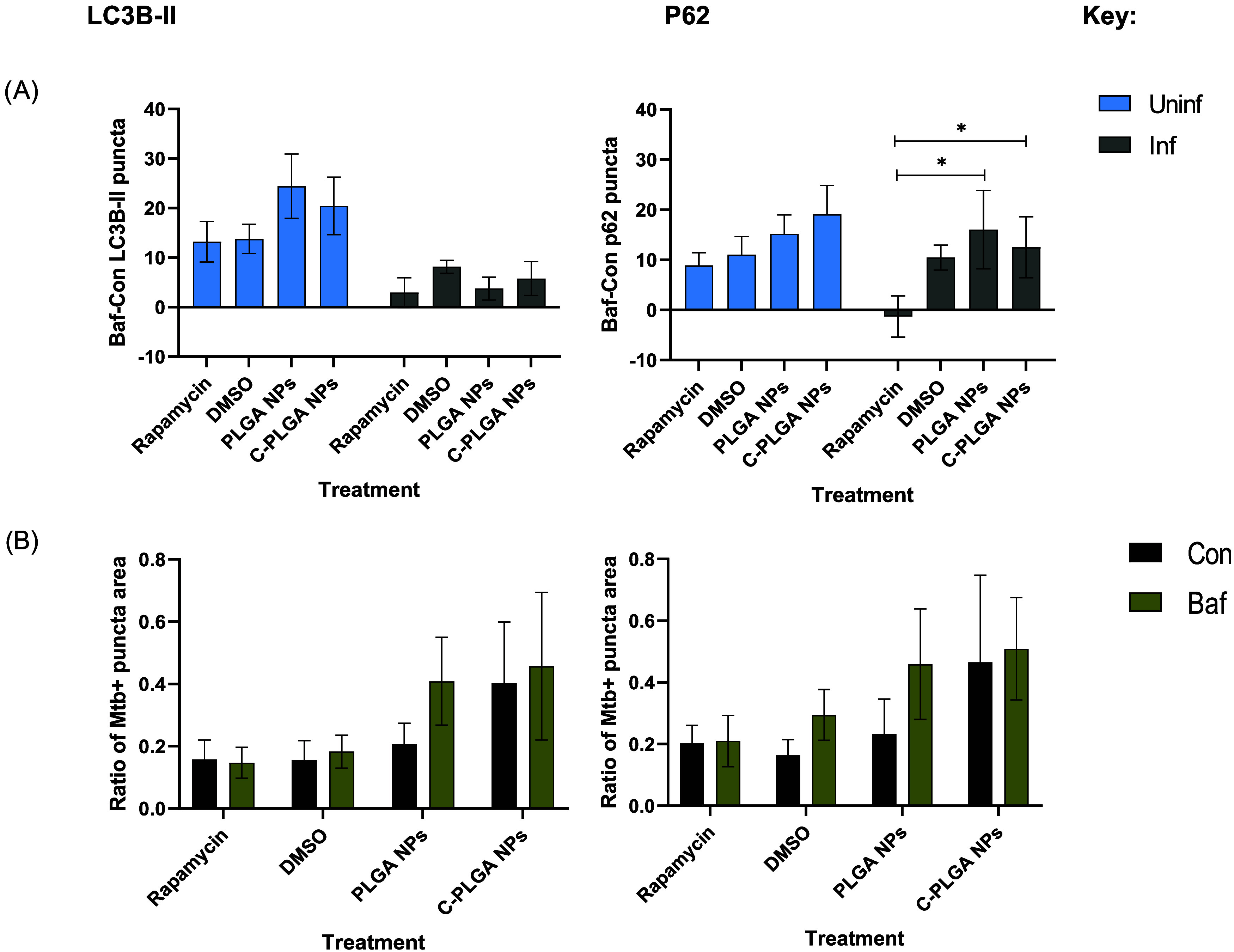
Quantitative analysis of autophagy induction in RAW 264.7 macrophages obtained by comparing the (A) Baf-Con of LC3B-II and p62 puncta in uninfected and infected RAW 264.7 macrophages and (B) the ratio of *M. tuberculosis*-positive LC3 and p62 puncta area, *n* = 3. (*) = *P* ≤ 0.05.

Upon encounter, pathogens are typically engulfed by the autophagic machinery.^19^ Phosphatidylethanolamine (PE)-conjugated LC3B (LC3B-II) is a marker and critical component of autophagosomes, which are double-membrane vesicles that engulf and degrade cytoplasmic components during autophagy in eukaryotes.^20^ LC3B-II is translocated to form part of the phagosomes and autophagosomes during autophagy, where it later becomes converted back to LC3B-I following lysosomal degradation. The autophagic receptor protein, p62, acts as adapter protein between the ubiquitinated cargo and LC3B-II on the autophagosomal membrane.^21^ Therefore, these markers were assessed to derive comprehensive autophagy data. Confocal microscopy showed successful staining of the targets LC3B-II and p62, in both the uninfected and *M. tuberculosis*-infected macrophages treated with and without bafilomycin (Figure 4A,B). Accumulation of green puncta in the bafilomycin-treated macrophages represented autophagosomes and/or autolysosomes with impaired lysosomal degradation due to increased lysosomal pH. Staining of both LC3B-II and p62 was seen in macrophages treated with dimethyl sulfoxide (DMSO) (negative control), rapamycin, as well as with PLGA NPs and C-PLGA NPs. Intracellular LC3B-II and p62 appear in the images as green spherical-like autophagy vesicles (punctate structures), for both uninfected and *M. tuberculosis*-infected macrophages. An increased number of LC3B-II and p62 puncta were identified in infected macrophages, with some puncta in close proximity to *M. tuberculosis*, as highlighted by the white arrowheads, Figure 4 A,B. The detection of autophagy through immunofluorescence microscopy verifies the results obtained using the CYTO-ID autophagy detection kit and validates the hypothesis that the NPs induce autophagy in both uninfected and *M. tuberculosis*-infected macrophages after 24 h of exposure. *M. tuberculosis* has been observed to impair autophagic turnover.^22^ Autophagic turnover was determined by calculating the difference in puncta count between the control and bafilomycin-treated samples. Enumeration of punctate structures Figure 4A,B, as well as the ratio of *M. tuberculosis*-positive puncta area of the intracellular *M. tuberculosis* with the autophagy vacuoles is shown in Figure 5. The PLGA NPs increased LC3B-II puncta per cell from 16.97 ± 4.31 in nonbafilomycin-treated macrophages to 41.56 ± 16.75 puncta per cell in bafilomycin-treated cells, while C-PLGA NPs increased the LC3B-II puncta per cell from 20.99 ± 7.97 in nonbafilomycin-treated macrophages to 42.17 ± 11.89 puncta per cell in the bafilomycin-treated macrophages (Figures 4A and 5). It was observed that the NPs induced autophagic turnover by increasing the amount of p62 puncta in the bafilomycin-treated *M. tuberculosis*-infected RAW 264.7 macrophages. PLGA NPs increased the amount of p62 puncta from 16.67 ± 6.46 in nonbafilomycin-treated macrophages to 32.71 ± 28.67 puncta per cell (*p*-value = 0.0120) in bafilomycin-treated RAW 264.7 macrophages. The C-PLGA NPs also increased the p62 puncta from 23.23 ± 12.11 in nonbafilomycin-treated macrophages to 35.74 ± 21.35 puncta per cell in bafilomycin-treated macrophages (Figures 4B and 5). No statistically significant differences were observed between the LC3B-II turnover or autophagic turnover induced by DMSO, PLGA and C-PLGA NPs when compared to the number of LC3B-II puncta induced by rapamycin in both infected and noninfected, bafilomycin-treated macrophages. However, the NPs demonstrated a significantly higher p62 autophagic turnover when compared with that of rapamycin in the *M. tuberculosis*-infected macrophages. When compared to the rapamycin turnover of 2.93 ± 8.56 puncta per cell, the PLGA NPs induced a significantly higher p62 turnover of 3.76 ± 6.56 per cell (*p*-value = 0.0145), whereas the C-PLGA NPs induced an even higher p62 turnover of 5.76 ± 9.67 per cell (*p*-value = 0.0495). On the other hand, no significant differences were found when comparing the ratio of colocalization of *M. tuberculosis* with LC3B-II or p62 puncta between infected macrophages treated with or without bafilomycin, after exposure to autophagy stimulants (Figure 5). The larger amount of both LC3B-II and p62 in the macrophages after treatment with bafilomycin was expected, as during autophagy, p62 becomes incorporated into the autophagosomal membrane by binding to LC3 and ubiquitin. This marker (p62) is normally degraded by the autophagy machinery but can also be used to monitor autophagosomal turnover as p62 has been proven to accumulate alongside ubiquitin when autophagy is inhibited.^23,24^

The ratios of *M. tuberculosis*-positive, LC3B-II puncta area were 0.158 ± 0.057, 0.156 ± 0.122, 0.206 ± 0.155, and 0.402 ± 0.518 for macrophages treated with rapamycin, DMSO, PLGA NPs, and C-PLGA NPs, respectively. The ratio of *M. tuberculosis*-positive, p62 puncta area were 0.203 ± 0.130, 0.156 ± 0.102, 0.233 ± 0.221, and 0.388 ± 0.560 for macrophages treated with rapamycin, DMSO, PLGA NPs, and C-PLGA NPs, respectively (Figure 5). These results therefore suggest that the autophagy induced by both LC3B-II and p62 captured intracellular *M. tuberculosis* inside the autophagosomes. The increase in p62 puncta after the addition of bafilomycin in the infected macrophages also suggests that these *M. tuberculosis*-containing autophagosomes mature into acidic autophagolysosomes that potentially degrade the captured bacterium.

### Nanoparticle Interaction and Uptake in Macrophages

**Figure 6:**
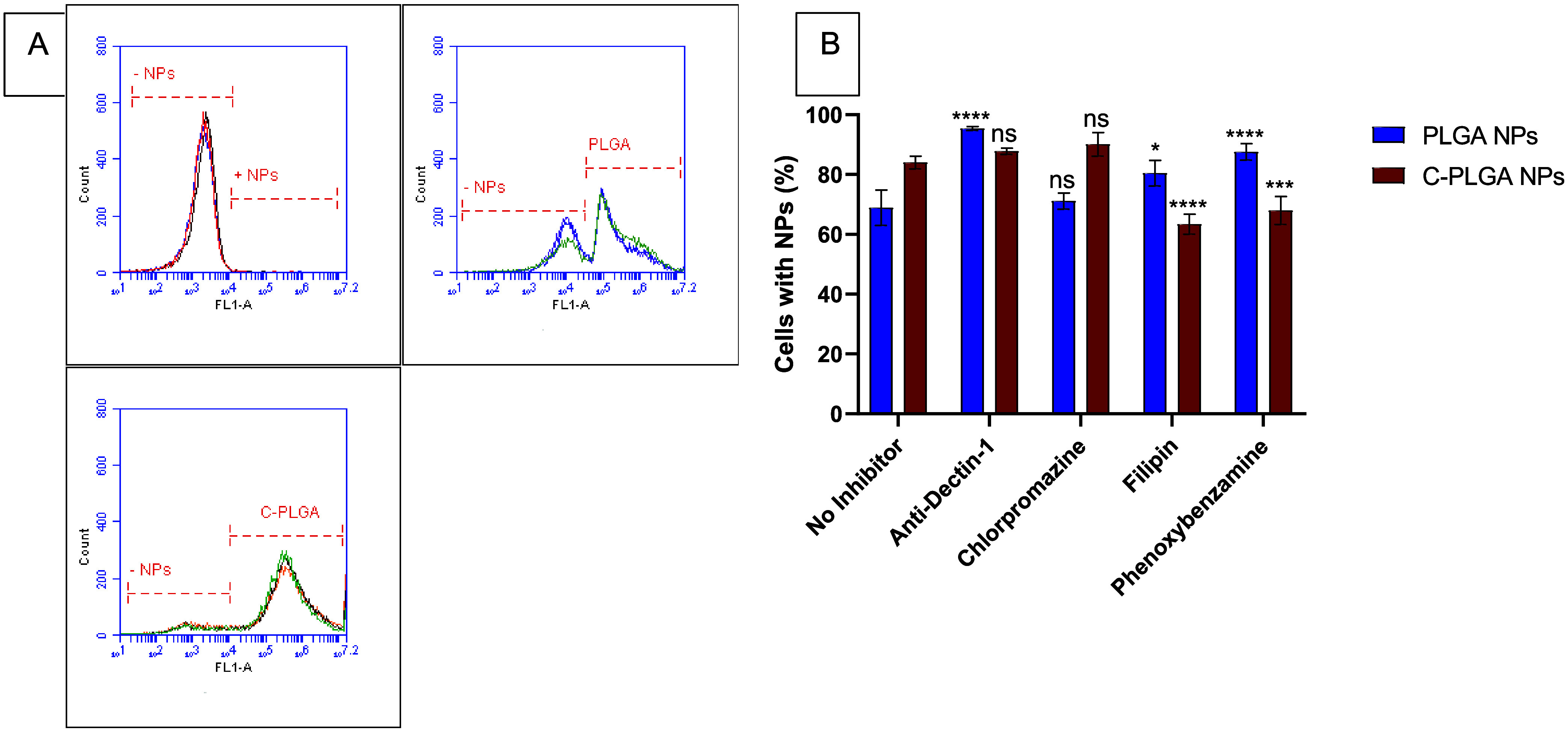
(A) Representative flow cytometry data of cellular uptake of PLGA and C-PLGA NPs by RAW 264.7 macrophages after 24 h of exposure (*n* = 3). (B) Comparative uptake of PLGA and C-PLGA NPs upon pretreatment with different inhibitors of various endocytosis pathways, i.e., an anti-Dectin-1 antibody, chlorpromazine, filipin, and phenoxybenzamine after 24 h of exposure (*n* = 3). ns = no significant difference (*P* > 0.05).

We assessed the mechanisms involved in the uptake of the NPs by macrophages and the extent of the role of Dectin-1 in the uptake of the NPs. In the absence of uptake inhibitors, we observed that C-PLGA NPs were taken up significantly more by the macrophages compared to PLGA NPs after 24 h of exposure (Figure 6A). Flow cytometry data indicated that the fluorescence intensity was 67.4 ± 5.1% and 83.8 ± 2.2% of cells with intracellular DiO-loaded PLGA NPs and C-PLGA NPs, respectively demonstrating significantly higher cellular uptake of C-PLGA NPs than PLGA NPs (p-value = 0.0015). Upon blocking the Dectin-1 receptor with anti-Dectin-1 antibodies, the amount of intracellular PLGA NPs significantly increased (93.2 ± 0.8% of macrophages with NPs; *p* = <0.0001) (Figure 6B). On the other hand, C-PLGA NPs showed no significant change in their uptake (87.9 ± 0.9%; *p* = 0.0389) upon the addition of the Dectin-1 receptor blocker (anti-Dectin-1 (Clec7a) monoclonal antibodies). The Dectin-1 receptor did not appear to influence the uptake of the C-PLGA NPs and instead is likely more involved in activation of the macrophages. Previously, we have shown basal production of phosphorylated ERK in THP-1 macrophages incubated with PLGA NPs, and enhanced production of phosphorylated ERK in cells incubated with C-PLGA NPs. The activation of ERK by the C-PLGA NPs was similar to that of Curdlan-only treated cells.^18^ Consequently, we chose to investigate further the uptake mechanism by the macrophages, focusing on endocytosis pathways such as phagocytosis and micropinocytosis.

In further investigation of the mechanism of uptake, macrophages (Figure 6B) were pretreated with chlorpromazine and filipin (inhibitors of clathrin-mediated endocytosis (CME) and caveolae-independent endocytosis (CIE), respectively). To determine whether the uptake was via phagocytosis or micropinocytosis, macrophages were pretreated with anti-Dectin-1 (Clec7a) monoclonal antibodies and phenoxybenzamine, respectively. Blocking of CME in macrophages was found to have no effect on the uptake of PLGA NPs (*p*-value > 0.9999) or C-PLGA NPs (*p* = 0.8750), suggesting that CME does not play a significant role in the uptake of both NPs (Figure 6B). NPs are generally known to be mainly taken up by cells using CME, primarily if they are positively charged, whereas negatively charged PLGA NPs are weakly taken up by CME and caveolin-mediated endocytosis independently.^25^ The inhibition of CIE on the other hand resulted in a significant increase in the uptake of PLGA NPs (*p* = 0.0275), while significantly reducing C-PLGA NP uptake by the macrophages (*p* < 0.0001). This suggested that CIE (possibly caveolae-mediated endocytosis) plays a significant role in the uptake of C-PLGA NPs but plays an insignificant role in the uptake of PLGA NPs in the macrophage. Lastly, blocking of micropinocytosis also significantly increased the uptake of PLGA NPs (*p* < 0.0001), while significantly reducing the uptake of C-PLGA NPs (*p* = 0.0007) in macrophages after 24 h of exposure, indicating that micropinocytosis plays a significant role in the cellular uptake of C-PLGA NPs but not in the uptake of PLGA NPs.

### Oropharyngeal Administration of the NPs is Nontoxic to Mice

**Figure 7:**
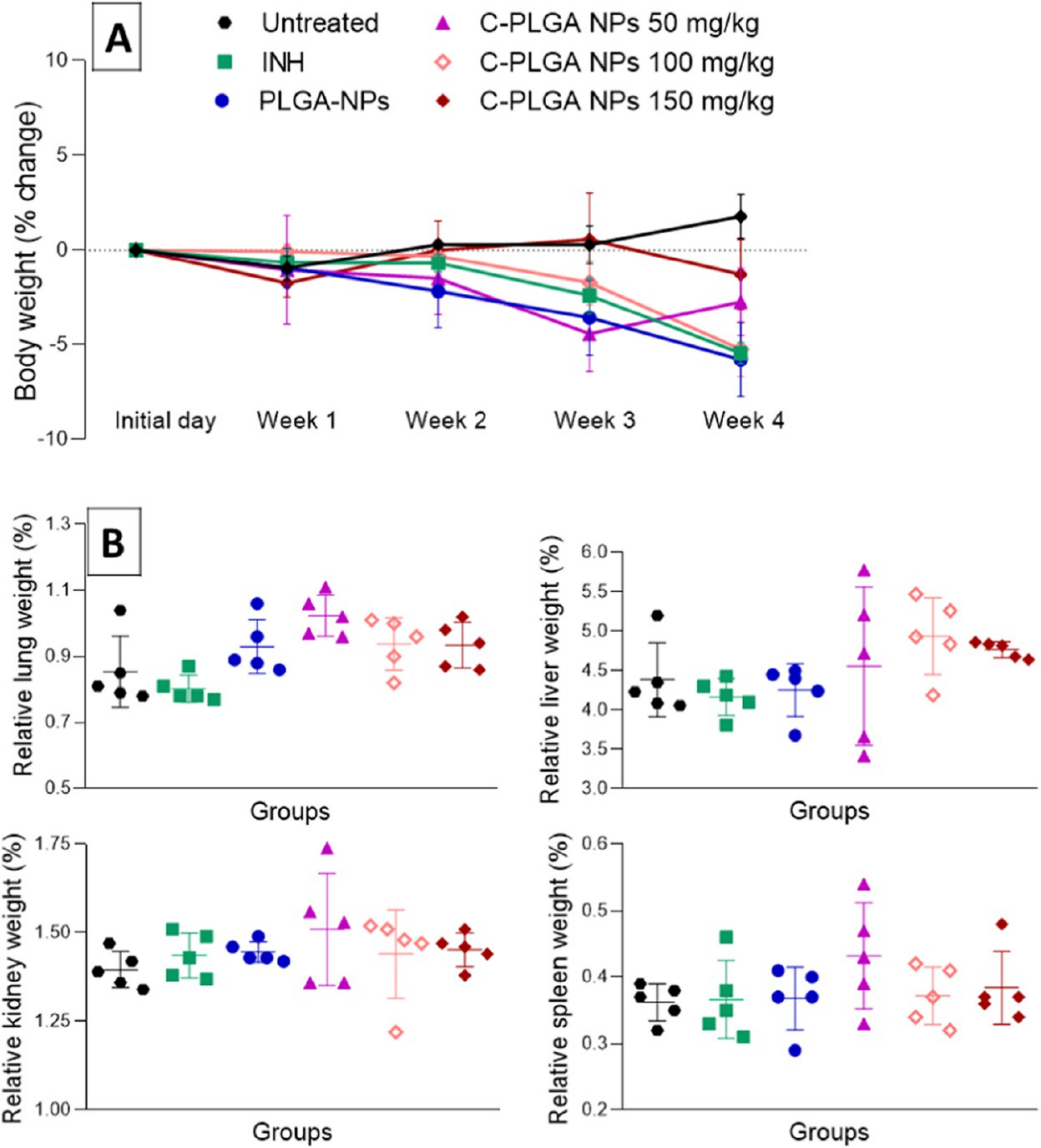
Percent body weight change of mice over 4 weeks of treatment, (A), for untreated (50 μL saline), isoniazid (INH) (25 mg/kg), PLGA NPs (100 mg/kg), C-PLGA NPs (50, 100, and 150 mg/kg). Day 1 represents the initial body weight before treatment. *n* = 5 and data expressed as mean ± standard error of the mean (SEM). Relative organ weight (lungs, liver, kidneys, and spleen) after 4 weeks of treatment calculated as a ratio by dividing the animal organ weight by its body weight and expressed as a percentage (B). *n* = 5 with data expressed as mean ± SD.

**Figure 8:**
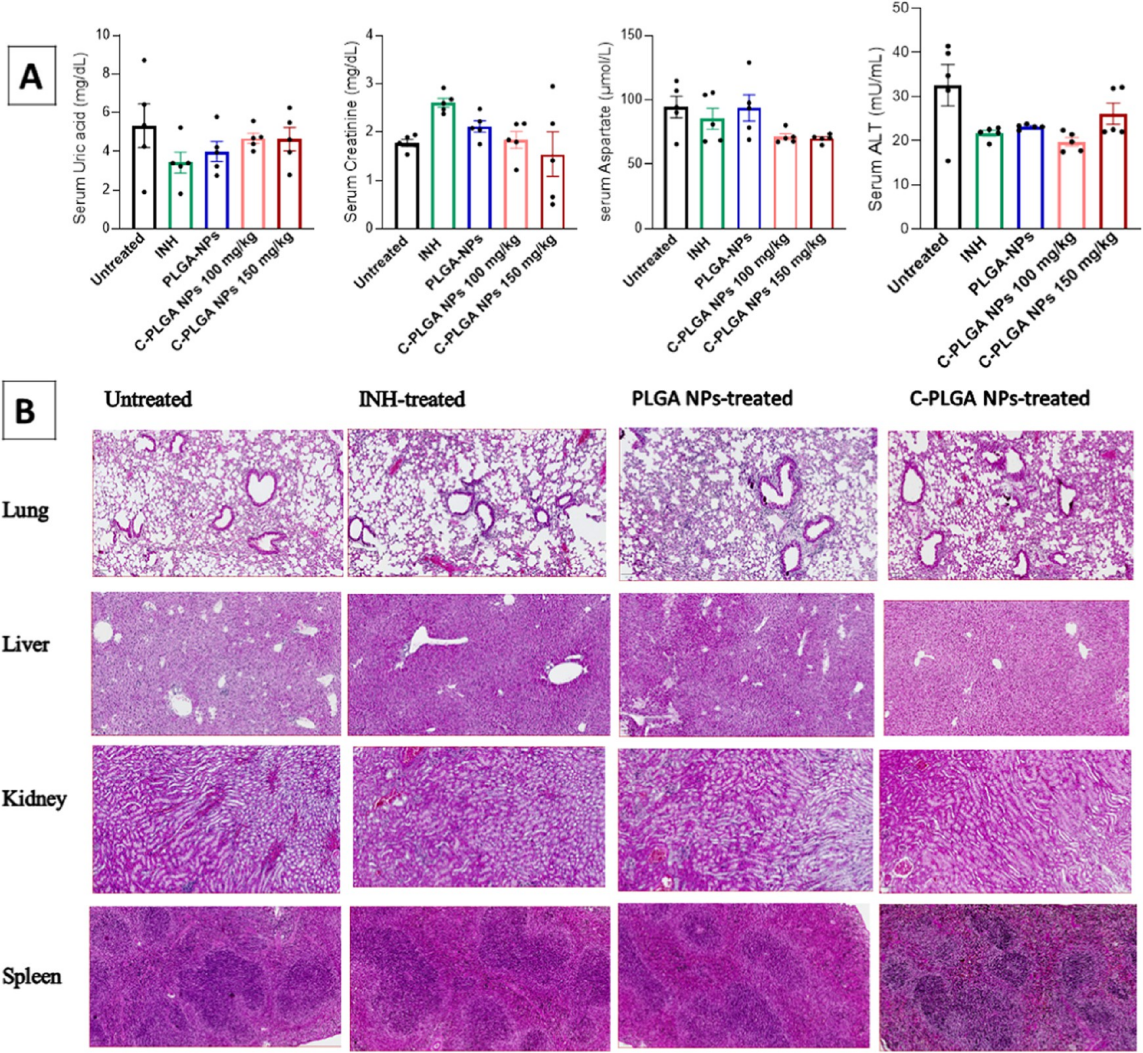
Biochemical analysis of blood serum is shown for untreated (saline), INH-treated (25 mg/kg), PLGA NP-treated (100 mg/kg), and the highest treatment doses of C-PLGA NP (100 and 150 mg/kg) (A) and data are expressed as mean ± SEM. Histopathology sections of hematoxylin and eosin (H&E) staining of the lungs, liver, kidneys, and spleen with original magnification 40×, sections displayed are representative of all organs from 5 mice per group and from one of two independent experiments, scale bar 100 μm (B).

We next assessed the toxicity of these NPs administered to healthy mice (C57BL/6) over 4 weeks. Mice were monitored daily with body weight measurements 3 times per week. No changes in physical appearance, general behavior, abnormal signs, or toxic effects and mortality were observed in all groups during the 4 weeks of treatment. Body weight expressed as percent change relative to the baseline weight is shown in Figure 7A. Changes in body weight with the highest administration doses (100 and 150 mg/kg per body weight) of C-PLGA NPs could be attributed to the physiological adaptation responses^26^ to the treatment rather than toxic effects. NP treatments did not influence the lungs, liver, kidneys, and spleen organ weights, and there were no significant differences (*p* > 0.05) when compared to the relative organ weights of untreated mice (Figure 7B). NPs also did not cause significant changes in uric acid, creatinine, aspartate, and alanine aminotransferase (ALT) compared to serum from untreated mice (Figure 8A). These observations were supported by histopathology analysis showing no evidence of inflammation/injury to the lungs, liver, kidneys, and spleen (Figure 8B).

### Oropharyngeal Administration of NPs to *M. tuberculosis*-Infected Mice Reduces Lung Bacterial Burden

**Figure 9:**
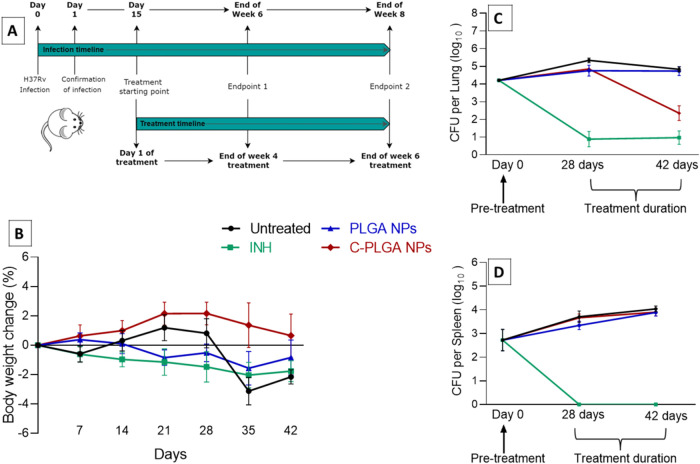
Experimental timeline of treatment of the infected mice (A). Trends of mice % body weight changes over 6 weeks of treatment (B). Bacterial burden in the lungs and spleen homogenates of mice infected with *M. tuberculosis* H37Rv and treated with INH, PLGA NPs or C-PLGA NPs administered via the oropharyngeal route (C, D). *n* = 7–10 mice. Untreated mice were administered with saline. Day 0 represents the bacterial burden at 2 weeks postinfection.

We next determined the efficacy of the NPs in *M. tuberculosis*-infected mice. Mice received treatments of either 150 mg/kg C-PLGA NPs or 100 mg/kg PLGA NPs or INH (25 mg/kg) or normal saline via the oropharyngeal route for 4 and 6 weeks (Figure 9A). 100 mg/kg of PLGA NPs was used, as it approximated the amount of PLGA present in the C-PLGA NPs. The presence of *M. tuberculosis* infection in mice has been associated with weight loss, often regarded as an indicative parameter of disease progression. Consequently, body weight was monitored in mice (Figure 9B) and we observed that none of the mice reached a moribund state or experienced more than a 15% reduction in body weight. Mice treated with C-PLGA NPs showed a modest weight gain of less than 3%, while the group receiving normal saline experienced the most pronounced weight reduction with a decrease of less than 3% (Figure 9B). *M. tuberculosis* burden in the lungs and spleens of mice was determined and expressed as log_10_ CFU per organ (Figure 9C,D). Upon infection with *M. tuberculosis* H_37_Rv and 2 weeks prior to the start of treatment, the initial mean log_10_ CFU/lung count was 4.21 ± 0.08, and the mean log_10_ CFU/spleen count was 2.72 ± 0.45.

After 4 weeks of treatment, there were no statistically significant differences in the lung bacterial load of mice treated with C-PLGA NPs (log_10_ CFU/lung 4.85 ± 0.09) or PLGA NPs (log_10_ CFU/lung 4.75 ± 0.31) in comparison to the group receiving normal saline (log_10_ CFU/lung 5.34 ± 0.13). However, the reduction in lung bacterial load was observed at 6 weeks of treatment with C-PLGA NPs (log_10_ CFU/lung 2.35 ± 0.41), and was statistically significant (*p* < 0.0001) in comparison to untreated mice receiving normal saline (log_10_ CFU/lung 4.83 ± 0.14) and PLGA NPs-treated mice (log_10_ CFU/lung 4.74 ± 0.26) as presented in Figure 9C. On the other hand, no reduction in spleen bacterial load was observed in the mice receiving the NP treatments (log_10_ CFU/spleen was 3.90 ± 0.16 and 3.89 ± 0.16 for C-PLGA NPs and PLGA NPs, respectively, after 6 weeks of treatment) in comparison to untreated mice (log_10_ CFU/spleen was 4.04 ± 0.12) as depicted in Figure 9D. INH could clear the burden in the lungs and spleen after 6 weeks (log_10_ CFU/lung was 0.97 ± 0.38 and complete clearance in the spleen). The outcomes of this section demonstrate that C-PLGA NPs treatment for 6 weeks results in a significant reduction of at least 2 log_10_ CFU in the lungs of mice infected with *M. tuberculosis* when compared against the vehicle control and mice treated with PLGA NPs. A previous study has demonstrated that subcutaneously administration of Curdlan alone in mice results in a significant reduction of *M. tuberculosis* burden in the lungs (*p* < 0.01) and spleen (*p* < 0.05).^27^ In our study, the NPs could reduce bacterial burden in the lungs but not in the spleen. This could imply that delivery of Curdlan through NPs using the oropharyngeal route has a more localized effect. It is possible that levels of the NPs reaching the spleen were not sufficient to significantly reduce the bacterial counts. Alternative administration routes may be necessary to achieve effective bacterial reduction in the spleen.

### Histopathology Analysis of Lung Tissue Following Treatment with NPs

**Figure 10:**
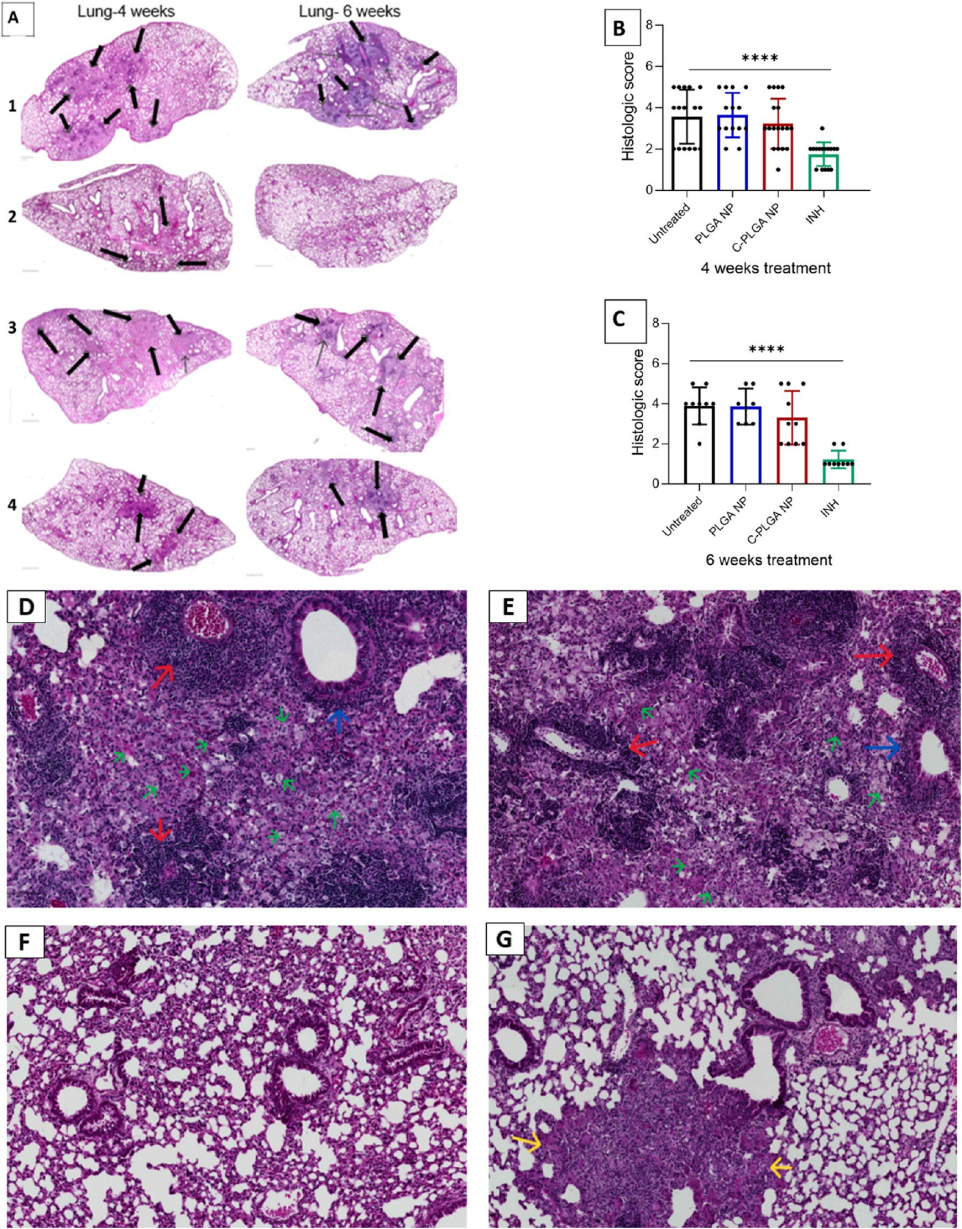
Histopathology sections of H&E staining of lung tissues from mice with original magnification 40× (Scale bar 100 μm). *M. tuberculosis*-infected control mice (A-1), mice treated with INH (A-2), PLGA NPs (A-3), 150 mg/kg C-PLGA NPs (A-4), and histopathological score (B, C) with 7–10 mice. The black arrows point to granulomas. Sections displayed are representative images of all organs from 7 to 10 mice per group. Section D to G represent the histopathological images of lung tissue at higher magnification (magnification = 7.85x, scale bar = 100 μm) after 6 weeks of treatment. Images show granuloma formation. Typical granulomas in untreated mice (D) and PLGA NP-treated mice (E) consisting of foamy vacuolated cells (green arrow) surrounding a lymphocytic core structure and containing infiltration of immune cells (red arrow = perivascular infiltration/inflammation and blue arrow = peribronchiolar infiltration/inflammation) such as lymphocytes. Normal lung tissue with few cell infiltration in INH-treated mice (F). Limited granulomatous inflammation (yellow arrow) mostly surrounded by normal lung tissue in C-PLGA NP-treated mice (G). Data expressed as mean ± SEM. The differences among groups were determined by analysis of variance (ANOVA) with a Dunnett’s multiple comparisons test (****P < 0.0001, *P* > 0.05, ns, nonsignificant).

We observed that lungs from mice treated with PLGA NPs for 4 weeks presented compact inflammatory lesions characterized by mononuclear infiltration with a large accumulation of lymphocytes within the epithelioid macrophages, similar to infected untreated mice (Figure 10A). After 6 weeks of treatment, lungs from mice treated with PLGA NPs showed findings similar to those observed in untreated mice, with lung tissue displaying prominent organized granulomas containing macrophages, lymphocytes, and increased numbers of foamy vacuolated cells (Figure 10D–G). In contrast, mice treated with C-PLGA NPs showed limited granulomatous inflammation with discrete lesions mostly surrounded by normal lung tissue after 6 weeks of treatment compared to untreated mice (Figure 10G). Semiquantitative analysis of lung tissue based on an image scoring system showed reduced inflammation with reduced granuloma size and number in the C-PLGA NPs-treated mice compared to untreated mice (Figure 10B,C)

### NPs Modulate Lung Cytokines and Chemokines in *M. tuberculosis*-Infected Mice

**Figure 11:**
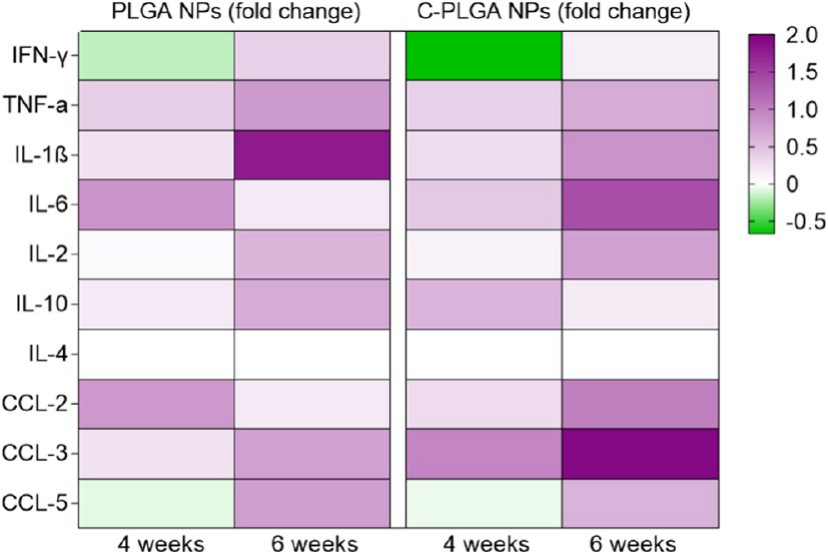
Cytokines and chemokine production from *M. tuberculosis*-infected mice at 4 and 6 weeks of treatment with NPs. Heat map representing changes in cytokine and chemokine levels in the *M. tuberculosis*-infected mice after 4 and 6 weeks of treatment with either PLGA or C-PLGA NPs. Each row of the heat map represents the fold change of each cytokine or chemokine as compared to untreated mice. Each column represents treatment periods. The color scale corresponds to the relative expression of the cytokine or chemokine with the minimum (below 0) and maximum (below 2) of all values.

We next conducted an analysis of the *in vivo* immunomodulatory effects of the Curdlan delivered via NPs in the *M. tuberculosis*-infected mice looking at various analytes, i.e., IL-1β, IL-2, IL-4, IL-6, IL-10, TNF-α, IFN-γ, CCL-2 (MCP-1), CCL-3 (MIP-1α) and CCL-5 (RANTES) (Figure 11) and Figure S1 INH was used as a positive control to validate the effective reduction of bacterial burden in mice. Consequently, our cytokines/chemokines analysis primarily focuses on comparing the C-PLGA NPs group with untreated mice and mice treated with NPs without Curdlan. After 4 weeks of treatment, IFN-γ levels were significantly lower in mice treated with C-PLGA NPs compared to untreated mice (*p* < 0.05). However, there was no statistically significant difference observed when compared to mice treated with PLGA NPs. Levels of TNF-α remained consistent across all three groups. The immune modulation of other cytokines did not show significant differences among mice treated with C-PLGA NPs, PLGA NPs, and untreated mice. After 6 weeks of treatment with C-PLGA NPs, IFN-γ and IL-10 levels showed a reduction compared to levels observed at 4 weeks but when compared to untreated mice, the decreases in IFN-γ and IL-10 levels at 6 weeks were similar. Levels of TNF-α in C-PLGA NPs group remain relatively the same as at 4 weeks. IL-1β levels remained relatively high when compared to untreated or INH-treated mice but significantly lower when compared to mice treated with PLGA NPs (*p* < 0.05). The levels of IL-4, IL-6, and IL-2 remained similar after 4 weeks and 6 weeks of treatment with C-PLGA NPs. The production of IL-4, IL-6, and IL-2 displayed no statistically significant differences between mice treated with NPs or untreated mice at both experimental endpoints. These data show that treating *M. tuberculosis*-infected mice with C-PLGA NPs for 6 weeks leads to the reduction of IFN-γ which may point to a shift toward a controlled and balanced immune response, therefore contributing to better treatment outcomes when compared to untreated or PLGA NPs-treated group. The activity of TNF-α, IL-6, and IL-1β suggest that C-PLGA NPs initially trigger an inflammatory response in the infected mice but later modulate the inflammation associated with the disease. The reduction implies a dampening of inflammation seen after the initial weeks of treatment. This could be a potential beneficial effect of C-PLGA NPs in controlling excessive inflammation, which is often detrimental to *M. tuberculosis* infection. IL-4 and IL-10 levels are generally low in uninfected C57BL/6 mice, and when exposed to *M. tuberculosis* infection, IL-4 production remains relatively low^28^ and the C57BL/6 mice produce minimal IL-10 during infection.^29^ Considering the differences in composition between PLGA NPs and C-PLGA NPs, the observed differences in the expression of cytokines such as IL-6 or IL-1β as shown in the heat map representing the fold change of cytokines and chemokines are likely due to the presence of Curdlan on the NPs (Figure 11). Since Curdlan binds to β-glucan receptors on myeloid cells like macrophages and DCs, and we have shown that C-PLGA NPs possess altered interaction and uptake in macrophages compared to PLGA NPs, therefore, there would subsequently be different immune response and cytokine expression. The distinct phenotypes observed after 4 weeks of treatment could be due to the dynamic nature of the immune response, which involves cell proliferation, immune activation, and functional changes that occur over time. As the infection progresses, T cells differentiate and other immune cells may be mobilized and activated, changing their behavior and function.

Chemokines CCL-2 (MCP-1), CCL-3 (MIP-1α), and CCL-5 (RANTES) coupled with proinflammatory cytokines such as IFN-γ and TNF-α, as well as the anti-inflammatory cytokine IL-10, are essential in limiting *M. tuberculosis* infections. Our data show that administering C-PLGA NPs to the infected mice significantly increased the level of CCL-2 in comparison to untreated mice (*p* < 0.01) and mice treated with PLGA NPs (*p* < 0.05) after 6 weeks of treatment (Figures 11 and S1). Additionally, the release of CCL-3 showed that mice treated with C-PLGA NPs had a statistically significant increase in CCL-3 from 4 weeks of treatment (*p* < 0.05 at week 4, *p* < 0.001 at week 6) compared to untreated mice. After 6 weeks of treatment, the release of CCL-3 was statistically significantly increased (*p* < 0.05) when compared to mice treated with PLGA NPs. The higher levels of CCL-2 and CCL-3 suggest that C-PLGA NP treatment contributes to the recruitment and migration of monocytes and macrophages to the sites of infection. However, the results showed that treating infected mice with C-PLGA NPs did not significantly affect the release of CCL-5 when compared to untreated mice or mice treated with PLGA NPs.

## Conclusions

*M. tuberculosis* is an intracellular pathogen that suppresses the antimicrobial response of the macrophage. We have shown that PLGA NPs and in particular C-PLGA NPs induce autophagy in *M. tuberculosis*-infected macrophages. The increase in p62 puncta after the addition of bafilomycin in the infected macrophages indicates that these autophagosomes mature to acidic autophagolysosomes that potentially degrade the captured bacterium. Oropharyngeal administration of C-PLGA NPs is effective in reducing the *M. tuberculosis* burden in the lungs of mice over 6 weeks of treatment. The C-PLGA NPs show dynamic modulation of cytokines and chemokines involved in immune cell recruitment. These findings provide new insights into host-directed therapies, which are stand-alone and not adjunctive treatments with coadministration of antibiotics. The treatment effect of the C-PLGA NPs was localized in the lungs, and there is an opportunity to develop and clinically evaluate an inhalable formulation of these NPs for the treatment of TB.

## Methods

### Nanoparticle Construction and Characterization

PLGA and C-PLGA NPs (8% w/w C-PLGA NPs) were synthesized and characterized as described in our previously published paper^16^ (Supporting Information, SI). To synthesize DiO-loaded PLGA and C-PLGA NPs, the organic phase was prepared by mixing 40 mg of C-PLGA dissolved in 400 μL of DMSO and 100 μg of DiO was dissolved in 3.6 mL of DCM at room temperature for 20 min, while using parafilm to prevent solvent evaporation and aluminum foil to protect the fluorescent DiO stain from light degradation. Thereafter, an organic-to-aqueous phase volume ratio (1:10) oil-in-water single emulsion was prepared by adding 3 mL of the organic phase (PLGA in DCM) dropwise to 30 mL of the aqueous phase (PVA in dH_2_O) (0.5% w/v) under probe sonication for 7 min, followed by evaporation of the solvent.

### Determination of Nanoparticle Autophagy Induction Using Fluorescence Microscopy

RAW 264.7 macrophages cultured in complete DMEM medium supplemented with 10% FBS and 1% Penicillin–Streptomycin (D10) treated with PLGA and C-PLGA NPs were evaluated for the capacity to induce autophagy using the CYTO-ID Detection Kit (ENZ-51031), (SI). The stained cells were immediately imaged with an Eclipse Ti-U fluorescence microscope at 40× magnification using the green FITC filter for imaging the autophagic signal and the blue DAPI filter to image the nuclear signal.

### Determination of Nanoparticle Autophagy Induction Using Flow Cytometry

RAW 264.7 macrophages treated with PLGA and C-PLGA NPs were analyzed for autophagy induction using the CYTO-ID Detection Kit (ENZ-51031), (SI). The CYTO-ID fluorescence data were then analyzed on the FlowJo v10.8.1 (BD Biosciences, Germany) software; first, the scatter plot population was gated to remove the cell debris and noninternalized NPs. The remaining macrophages were then gated to remove doublets by changing the *Y*-axis from a side-scatter area (SSC-A) to a forward scatter-width setting in the *x*-axis (FSC-W vs FSC-A), leaving only single cells. The single cells were then gated and measured for fluorescence while keeping at least 1% of the untreated macrophages in the gate using the FSC-W vs FITC-A settings to compensate for the autofluorescence of the macrophages. Triplicated independent experiments were conducted for each sample using 10,000 events per sample.

### *M. tuberculosis* Infection of Macrophages and Autophagy Quantification Using Confocal Microscopy

RAW 264.7 macrophages were prepared as previously described^30,31^ (SI). Macrophages were incubated for 4 h at 37 °C and 5% CO_2_ to allow phagocytosis of bacteria by the cells. To remove extracellular bacteria, macrophages were subsequently washed twice with D10 medium, and the infection was permitted to continue for a total of 48 h. At the 24 h time point, cells were treated with PLGA NPs, C-PLGA NPs, the DMSO vehicle control, or rapamycin for 24 h. Rapamycin was added as an additional treatment known to induce autophagy in THP-1 macrophages.^32^ Corresponding experiments were also conducted for uninfected macrophages, with treatments and fixation performed in the same manner. For each treatment, macrophages were exposed to bafilomycin A1 (Sigma-Aldrich, MO) at a final concentration of 100 nM for 3 h before the fixation of cells. Bafilomycin is used to inhibit lysosomal protein degradation, resulting in autophagic turnover or a buildup of autophagosomes.^33^

### Immunofluorescence Sample Preparation and Image Acquisition

To examine markers LC3B-II and p62, after exposure to bafilomycin, cells were then incubated overnight with LC3B (Ab51520, Abcam) and p62 (Ab91526, Abcam) antibodies diluted in 0.3% bovine serum albumin (BSA) in phosphate-buffered saline (PBS). Details of the protocol are given in Supporting Information.

### Determination of Uptake of DiO-Loaded PLGA and C-PLGA NPs Using Fluorescence Microscopy

To perform qualitative and quantitative investigations on the uptake of DiO-loaded PLGA and C-PLGA NPs, RAW 264.7 macrophages were grown to confluency. Thereafter, cells were seeded at a density of 100,000 cells/mL and allowed to attach overnight on a 12-well plate. Macrophages were then washed twice with PBS at pH 7.5, to remove any unattached cells. The attached cells were then subsequently treated with the DiO-loaded C-PLGA NPs dissolved in D10 at a final NP concentration of 0.5 mg/mL, for 24 h (SI).

### Determination of the Uptake of DiO-Loaded PLGA and C-PLGA NPs Using Flow Cytometry

To investigate possible pathways of PLGA and C-PLGA NP uptake, RAW 264.7 macrophages were used. To investigate whether the NPs were taken up via CME or CIE, the attached cells were independently pretreated for 30 min with the medium containing 10 μg/mL chlorpromazine and 5 μg/mL filipin, respectively.^34,35^ To investigate whether the uptake was via phagocytosis or micropinocytosis, the macrophages were pretreated with 3 μg/mL of anti-Dectin-1 (Clec7a) monoclonal antibodies and 5 μM of phenoxybenzamine and incubated at 37 °C for 2 and 1 h, respectively.^36−38^ The pretreated macrophages were then washed three times with PBS treated with the DiO-loaded PLGA and C-PLGA NPs dissolved in D10 at a final NP concentration of 0.5 mg/mL, for 24 h (SI).

### Animal Studies

All animal experiments and protocols of this study were conducted with ethical clearances approved by the UCT Animal Research Ethics Committee (018-040) and the University of the Western Cape Animal Research Ethics Committee (AR19/9/2). Female mice C57BL/6j aged between 8 to 10 weeks old were purchased from the Research Animal Facility (RAF) of UCT. Mice were maintained under specific pathogen-free conditions in the Biosafety Level 3 facility of the RAF unit in the standardized environment (22 ± 2 °C, 55% ± 15% humidity) on a 12 h light/12 h dark cycle. The cages were supplied with drinking water and sterilized formulated food pellets. A red mouse house was provided in each cage with the appropriate bedding and nesting material. An acclimatization period of 7 days was used prior to toxicity and efficacy experiments.

### Toxicity Study on Healthy Mice

Mice were randomly assigned into groups consisting of 5 mice each: C-PLGA NPs treatment groups at various doses (50, 100, and 150 mg/kg of body weight), PLGA NPs treatment group (100 mg/kg of body weight), and INH treatment group (25 mg/kg of body weight). The control group received saline (50 μL/mouse). Each dose was administrated via the oropharyngeal route, three times per week for 4 weeks. The body weight was recorded and mice were monitored (SI). On day 29, mice were euthanized, and the blood was drawn by cardiac puncture and placed into 1.5 mL microtubes. The test tubes were left at room temperature to clot, followed by centrifugation at 10,000 rpm for 10 min to collect the blood serum. The blood serum was kept at −80 °C until use for biochemical analysis. Effects of C-PLGA NP treatments on kidney and liver function were assessed by quantifying aspartate, ALT, creatinine, and uric acid and compared with the saline control and PLGA NP groups. Liver and kidney assessments were also performed on samples from the INH group. All kits were purchased from Sigma-Aldrich and assays were conducted using the POLARstart Omega (BMG LABTECH) microplate reader. Lungs, liver, kidneys, and spleen were collected. The organ weights were recorded, and organs were stored for histopathology analysis.

### Animal Infection and Treatment with NPs

Mice were challenged with *M. tuberculosis* H37Rv via the intranasal route. The pulmonary infection dose was confirmed by euthanizing five mice 24 h postinfection. Another group of four mice was euthanized 14 days postinfection to assess the bacterial load prior to treatment. The remaining mice were randomly assigned into 4 groups: C-PLGA NPs (150 mg/kg), PLGA NPs (100 mg/kg), INH (25 mg/kg), and untreated group (50 μL of saline/mouse) with 7–10 mice per group per experimental endpoint (Figure 9A). Each dose was administrated via the oropharyngeal route, three times per week for 4 and 6 weeks. The body weight was recorded and mice were monitored. Mice were euthanized after 4 weeks of treatment (experimental endpoint 1) and 6 weeks of treatment (experimental endpoint 2). At each endpoint, the lungs, liver, kidneys, and spleen were collected and their weights were recorded. These organs were kept for histopathological analysis. Also, a portion of the lungs and spleen were homogenized for CFU analysis and cytokine/chemokine Luminex assay.

### CFU Determination in Organs

Animals were euthanized after 4 and 6 weeks of treatment, which correspond to 6 and 8 weeks postinfection, respectively. Bacterial loads from the harvested lungs and spleens were quantified by homogenizing each tissue in sterile saline 0.9% (w/v) sodium chloride solution containing 0.04% (v/v) Tween-80 using a glass tissue homogenizer. Homogenates were plated in duplicate (100 μL on each side) in 10-fold serial dilutions onto plates containing Difco Middlebrook 7H10 agar media supplemented with 0.5% glycerol and 10% OADC. Plates were incubated for 21 days at 37 °C, followed by a manual count of mycobacterial colonies grown on agar plates. CFU count was calculated as CFU/organ and expressed as log_10_ for each experimental endpoint.

### Histopathology

Briefly, organ fixation was carried out immediately upon harvesting the organ by immersing the organs in vials containing 10% v/v formalin in PBS followed by staining of organ sections with hematoxylin and eosin (H&E) dye. The microscopic photographs were captured on Grundium Ocus40 scanner MGU-0003, numerical aperture: 0.75, resolution: 10 μm/pixel, image sensor: 12 Middle brook. To assess the lung inflammation and tissue damage, images were graded for severity by analyzing multiple random fields in 3 sections of each tissue per mouse. Based on the extent of granulomatous inflammation in the lungs, each mouse was assigned a histological score as previously described,^39^ score 0 = no lesion, score 1 = minimal lesion (1–10% tissue area affected in section involved), score 2 = mild lesion (11- 30% area affected), score 3 = moderate lesion (31–50% area affected), score 4 = marked lesion (51- 80% area affected), score 5 = severe lesion (more than 80% area affected).

### Quantification of Cytokines and Chemokines

Lung and spleen homogenates were centrifuged at 10,000 rpm for 10 min at 4 °C, and the supernatants were filtered using sterile 0.22 μm syringe filters. A customized Marked Mouse Cytokine and Chemokine Magnetic Bead Panel (cytokine/chemokine analytes with product ID MCYTOMAG-70k) was purchased from Merck (Merck Life Sciences, Germany). Cytokines IL-1β, IL-2, IL-4, IL-6, IL-10, TNF-α, IFN-ϒ, and chemokines CCL-2 (MCP-1), CCL-3 (MIP-1α), CCL-5 (RANTES) from supernatants were quantify by multiplex Luminex assay (SI) and the plate was read on a Bio-Plex reader (Bio-Plex TM, Bio-Rad Laboratories) to quantity the analytes.

## Statistical Analysis

All experiments were conducted in triplicate, the data, expressed as mean ± standard deviation, were interpreted using GraphPad Prism 8 (GraphPad Software). A one-way ANOVA and two-way ANOVA tests were applied to determine the significance of any differences between the means. Differences were considered significant if *p*-values were ≤0.05. To minimize bias in the animal experiments, all researchers and laboratory technicians involved in histopathology, CFU determination, and cytokines/chemokines quantification were blinded to the experimental conditions (except the principal investigator).

## Data Availability

Data and material requests should be addressed to Admire Dube (adube@uwc.ac.za).

## Supporting Information Available

Cytokines and chemokines production from *M. tuberculosis*-infected mice after 4 and 6 weeks of treatment (Figure S1); detailed materials and methods (PDF)
