# Supplementary material for: Immunomodulatory Nanoparticles Induce Autophagy in Macrophages and Reduce Mycobacterium tuberculosis Burden in the Lungs of Mice
Source: ACS Infect Dis. 2025 Feb 25;11(3):610–25. doi: 10.1021/acsinfecdis.4c00713 (PMC11915374; doi:10.1021/acsinfecdis.4c00713)
Supplement: Supplementary file 1 — id4c00713_si_001.pdf [file id4c00713_si_001.pdf]

SUPPORTING INFORMATION

An immunomodulatory nanoparticle induces autophagy in macrophages and reduces *Mycobacterium tuberculosis* burden in lungs of mice

Raymonde B. Bekale[1], Retsepile E. Maphasa[1], Sarah D'Souza[1], Nai Jen Hsu[2,3,4], Avril Walters[2,3,4], Naomi Okugbeni[6,7], Craig Kinnear[6,7], Muazzam Jacobs[2,3,4, 5], Samantha L. Sampson[6], Mervin Meyer[8], Gene D. Morse[9], Admire Dube[1*]

[1]Infectious Disease Nanomedicine Research Group, School of Pharmacy, University of the Western Cape, Cape Town, South Africa
[2]Division of Immunology, Department of Pathology, University of Cape Town, South Africa
[3]National Health Laboratory Service, Cape Town, South Africa
[4]Neuroscience Institute, University of Cape Town, Observatory, South Africa
[5]Institute of Infectious Disease and Molecular Medicine, Faculty of Health Sciences, University of Cape Town, Observatory, South Africa
[6]DSI-NRF Centre of Excellence for Biomedical Tuberculosis Research; South African Medical Research Council Centre for Tuberculosis Research; Division of Molecular Biology and Human Genetics, Faculty of Medicine and Health Sciences, Stellenbosch University, Cape Town, South Africa
[7]South African Medical Research Council Genomics Platform, Tygerberg, South Africa
[8]Department of Science and Innovation/Mintek Nanotechnology Innovation Centre, Biolabels Node, Department of Biotechnology, University of the Western Cape, Cape Town, South Africa
[9]Center for Integrated Global Biomedical Sciences, School of Pharmacy and Pharmaceutical Sciences, University at Buffalo, State University of New York, Buffalo, New York, USA

These authors contributed equally: Raymonde B. Bekale, Retsepile E. Maphasa

* Email: adube@uwc.ac.za

Detailed materials and methods

Materials. Curdlan from Alcaligenes faecalis, poly vinyl alcohol (PVA: MW, 13–23 kDa, partially hydrolysed (98%)), Resomer RG503H poly(D,L-lactide-co-glycolide) (50/50) with the terminal carboxylic acid (24–38 kDa) and non-acid terminated PLGA (MW, 30–60 kDa) were purchased from Sigma-Aldrich (St Louis, MO). All reagents were of analytical grade or above and were used as purchased unless otherwise stated. Deionised water was obtained from a Barnstead EasyPure (II) UV-ultrapure water system (Thermo Fisher Scientific, USA) and was used throughout the study. RAW264.7 murine macrophages (ATCC TIB-71) were cultured in D10 (Dulbecco's modified Eagle's medium (DMEM) supplemented with 10% heat-inactivated Fetal Bovine Serum (FBS) both of which were obtained from Thermo Fisher), with passage number maintained below 30. Liquid mycobacterial cultures, M. tuberculosis ΔleuDΔpanD, M. tuberculosis ΔleuDΔpanD::pMV306hsp+lux, Table 1, were grown in 7H9-OGT – 7H9 (Becton Dickinson), supplemented with 10% oleic acid–albumin–dextrose– catalase (OADC; Becton Dickinson, NJ, United States), 0.2% (v/v) glycerol (Sigma-Aldrich), 0.05% (v/v), Tween-80 (Sigma-Aldrich). Solid cultures were grown on 7H10 agar (Becton Dickinson, NJ). Additional supplements included pantothenate (24 µg/mL), leucine (50 µg/mL), hygromycin (50 µg/mL) (Invitrogen) and kanamycin (25 µg/mL) (Sigma-Aldrich, MO), the latter for maintenance of the pMV306hsp+lux plasmid. Phosphate-Buffered Saline (PBS) was obtained from Thermo Fisher and the MTT (3- (4,5-dimethylthiazol-2-yl)-2,5-diphenyltetrazolium bromide) together with the DMSO solution was purchased from Sigma-Aldrich. Secreted cytokines were analysed using a customized mouse Multiplex R&D luminex kit (Thermo Fisher).

Nanoparticle construction and characterization. PLGA and C-PLGA NPs (8% w/w CPLGA NPs) were synthesized and characterized as described in our previously published paper [1]. Briefly, an aqueous solution was prepared by dissolving 5 mg/mL of PVA in deionized water. The organic solution was prepared by adding 24 mg of 10% w/w C-PLGA copolymer and 6 mg of PLGA to a mixture of DMSO/DCM (1:9). 3 mL of the organic solution was added dropwise into 30 mL of aqueous solution under probe sonicator for 6 min, followed by evaporation for less than 1 h and centrifugation at 4800 x g for 20 min.

The collected pellet was washed twice with deionized water. As a cryoprotectant, a 1% w/v sucrose solution was prepared and added to the final volume of NPs re-dispersed in a ratio of 1:2 (sucrose solution to 8% w/w C-PLGA NPs) and then frozen at -80°C prior to lyophilization over a period of 3 days. The same protocol was used to synthesize PLGA NPs in which the organic phase was prepared by dissolving 30 mg of PLGA in a mixture of DMSO and DCM.

The physico-chemical properties of the formulated PLGA NPs and 8% w/w C-PLGA NPs were analysed in de-ionised water and 0.9% w/v NaCl solution using DLS techniques for size, size distribution and zeta potential. SEM analysis was performed for size, shape, and morphology. To prepare the samples to be analysed, freeze-dried NPs were mounted to a sample holder with double-sided carbon tape and carbon coated. The morphology and size of the NPs were then analysed with a Tescan Mira SEM (TESCAN Mira3 XMU, USA) and EDS was performed with the Thermo Fisher Nova NanoSEM230 using an Oxford X-max detector and INCA software. SEM images were processed with the Digimizer image analysis software® (version 5.7.2 from MedCalc Software Ltd, Belgium) to obtain the mean size of the NPs.

Determination of nanoparticle autophagy induction using fluorescence microscopy. RAW 264.7 macrophages cultured in complete DMEM medium supplemented with 10% FBS and 1% Penicillin-Streptomycin (D10) treated with PLGA and C-PLGA NPs were evaluated for the capacity to induce autophagy using the CYTO-ID Detection Kit (ENZ-51031). Briefly, the macrophages were grown to 70% confluency and seeded on Greiner 12 well plate at a density

of 100 000 cells/ml overnight. The cells were then washed twice with PBS and treated with 1 mg/ml of PLGA or C-PLGA NPs prepared in D10, using untreated cells as a negative control and serum starved cells as a positive control for 24 h. Post treatment, the medium was removed, and cells were washed twice with 1x Assay buffer containing 5% FBS. 100 µl of microscopy dual reagent, consisting of the CYTO-ID green detection reagent and the Hoechst 33342 nuclear stain, was then added to cover each well of monolayer cells. The cells were protected from light by covering the plate with aluminium foil, and incubated for 30 min at 37°C. The cells were then carefully washed with 100 µl of 1x Assay buffer, excess buffer was removed, and the cells were fixed by incubating with 4% formaldehyde and washing 3 times with 1x assay buffer. The stained cells were then immediately imaged with an Eclipse Ti-U fluorescence microscope at 40x magnification using the green FITC filter for imaging the autophagic signal and the blue DAPI filter to image the nuclear signal.

Determination of nanoparticle autophagy induction using flow cytometry. RAW 264.7 macrophages treated with PLGA and C-PLGA NPs were analysed for autophagy induction using the CYTO-ID Detection Kit (ENZ-51031). Briefly, the RAW 264.7 macrophages were grown to 70% confluency and seeded on 12 well plates at a density of 100 000 cells/ml overnight, at 37°C, 5% CO_2. The cells were then washed twice with PBS and treated with 1 mg/ml of PLGA and C-PLGA NPs prepared in D10 medium, using untreated cells as a negative control and serum starved cells as a positive control for 24 h. Post treatment, the medium was removed, and cells were washed twice with 1x Assay buffer containing 5% FBS. Cells were then lifted from the wells by adding 350 µl of trypsin and transferred to 1.5 ml Eppendorf tubes, followed by centrifugation at 1000 rpm for 5 min to pellet the cells. Cells were washed by resuspending the cell pellet in cell culture medium and collecting the cells by centrifugation. The cells were resuspended in 250 µl of indicator free cell culture medium containing 5% FBS. 250 µl of diluted CYTO-ID green stain solution was added to each sample and mixed well by gentle pipetting. Cells were incubated for 30 min at 37°C in the dark, and the cells were collected by centrifugation and washed with 1x assay buffer. Cells pellets were then resuspended in 500 µl of 1x assay buffer and analysed with a BD FACSAria™ III flow cytometer (BD Bioscience, Germany) using 10 000 events per sample, the fluorescence readings of NPs treated and non-treated RAW 264.7 macrophages were measured using the FITC-A channel.

The CYTO-ID fluorescence data was then analysed on the FlowJo v10.8.1 (BD Biosciences, Germany) software, first the scatter plot population was gated to remove the cell debris and non-internalized NPs. Remaining macrophages were then gated to remove doublets by changing the Y-axis from a side scatter-area (SSC-A) to forward scatter-width versus a forward scatter-area setting in the x-axis (FSC-W vs FSC-A), leaving only single cells. The single cells were then gated and measured for fluorescence while keeping at least 1% of the untreated macrophages in the gate using the FSC-W vs FITC-A settings to compensate for the autofluorescence of the macrophages. Triplicated independent experiments were conducted for each sample using 10 000 events per sample.

Bacterial cell culture for in vitro studies. The *M. tuberculosis* strain, H37Rv (BEI Resources, USA), was used for all macrophage infection experiments. This strain was formerly transformed with an *mCherry*-expressing plasmid (Plasmid #24659, Addgene, UK) by Victoria Cole, and a Biosafety Level III (BSL3) laboratory was used for all culturing and infection experiments. Infected samples were only removed from the facility after fixing with 4% paraformaldehyde (Alfa Aesar, Germany) for one hour to inactivate the bacteria.

Culturing and freezing bacterial stocks. *M. tuberculosis* bacterium was grown in 1:25 ratio of 7H9 medium (Becton-Dickinson, USA) supplemented with 10% Oleic Albumin Dextrose Catalase (OADC) (Becton-Dickinson), 0.2% glycerol, 0.05% Tween 80 and 50 µg/ml Hygromycin under 37°C incubation while shaking at 180 rpm. Bacterial cultures used for infection experiments were grown in medium devoid of Tween 80, and stock of the *M. tuberculosis* bacterium with Optical Density at 600 nm (OD_{600}) of approximately one was collected and frozen described [2, 3].

Infection of macrophages with *M. tuberculosis*. RAW 264.7 macrophages were centrifuged for 10 min at 4000 rpm and resuspended in D10 medium devoid of antibiotics. The frozen stocks of *M. tuberculosis* were grown in Tween-free medium to an OD_{600} of 0.5 -0.8 (log phase). The *M. tuberculosis* bacterium was passed 20 times through a 21 G needle, and 10 times through a 25 G needle, to achieve single bacterial cells. The bacterial cultures were subsequently passed through a 5.0 µm filter and the optimal density of the resulting filtrates were measured using a spectrophotometer [2, 3]. The filtrate was then diluted to the required concentration and exposed to the macrophages as a monolayer suspension resulting in a Multiplicity of Infection (MOI) of two bacilli to one host cell. Macrophages were then incubated for 4 h at 37^0C and 5% CO_2 to allow phagocytosis of bacteria by the cells. To remove extracellular bacteria, macrophages were subsequently washed twice with D10 medium and the infection was permitted to continue for a total of 48 h. At the 24 h time point, cells were treated with PLGA NPs, C-PLGA NPs, DMSO vehicle control, or rapamycin for 24 h. Rapamycin was added as additional treatment known to induce autophagy in THP-1 macrophages [4]. Corresponding experiments were also conducted for uninfected macrophages with treatments and fixation performed in the same manner. For each treatment, macrophages were exposed to bafilomycin A1 (Sigma-Aldrich, MO) at a final concentration of 100 nM for 3 h before the fixation of cells. Bafilomycin is used to inhibit lysosomal protein degradation, resulting in autophagic turnover or a build-up of autophagosomes [5].

Immunofluorescence sample preparation and image acquisition. To examine markers LC3B-II and p62, after exposure to bafilomycin, RAW 264.7 macrophages were washed thrice with PBS and fixed with 4% paraformaldehyde solution in PBS at room temperature for one hour. Thereafter, 8-well chambers were carefully removed from the BSL3 facility, and the cells were washed for 10 min with PBS and subsequently permeabilized with 0.2% Triton-X 100 in PBS for 10 min at room temperature. Cells were then washed in PBS and blocked for 2 h with 3% BSA in PBS at room temperature. Cells were then incubated overnight with LC3B (Ab51520, Abcam) and p62 (Ab91526, Abcam) antibodies diluted in 0.3% BSA in PBS. The RAW 264.7 macrophages were subsequently washed with 0.05% Tween 20 in PBS for 30 min to remove nonspecific bound antibody, followed by incubation with Alexa-Fluor 488 conjugated Anti-Rabbit antibody (Ab150077, Abcam) at room temperature for 90 min in the dark. The macrophages were thereafter washed for 30 min with 0.05% Tween 20 in PBS and incubated for 10 min with Hoechst nuclear stain (#33342, Thermo Fisher Scientific) at room temperature. After nuclear staining, the macrophages were washed with PBS and preserved by mounting with DAKO mounting medium (Agilent).

A Carl Zeiss (Oberkochen, Germany) confocal microscope was utilized to perform fluorescence-based imaging, using a Plan-Apochromat 63x/1.4 Oil DIC M27 objective to acquire the raw image stacks/series. The emission detection was achieved using 405 nm, 488 nm, 561 nm, and 633 nm lasers with a GaAsP detector as light sources. To achieve an optimal signal/noise ratio with low pixel saturation, the master gain and laser power were selected for 408 nm (Hoechst), 488 nm (Alexa-Fluor 488) and 561 nm (Auramine-rhodamine). The track filters were set as 410-497 nm for Hoechst, 499-579 nm for Alexa-Fluor 488, and 579-641 nm

for Auramine-rhodamine. The images produced were used to determine the presence and colocalization of autophagy punctate structures and intracellular fluorescent *M. tuberculosis* before and after the addition of bafilomycin. The number of punctate structures was subsequently counted and used to calculate the autophagic turnover values by subtracting the control puncta values from the bafilomycin values. The number of cells imaged for each condition are denoted in Table 1. In further analysis, *M. tuberculosis* bacilli was converted to a mask and used as a Region of Interest to enumerate LC3B-II and p62 puncta in close proximity of the bacteria. The ratio of *M. tuberculosis* + puncta was then calculated as follows:

$$\frac{\text{Area of LC3B−II or p62 puncta overlaying } \textit{M.tuberculosis}}{\text{Area of } \textit{M.tuberculosis}}.$$

Table S1: Number of cells imaged for each condition

**LC3B-II**	Uninf Con	Uninf Baf	Inf Con	Inf Baf
Rapamycin	345	350	239	261
DMSO	235	219	309	233
.PLGA	261	206	175	189
C-PLGA	247	228	180	171
**p62**	Uninf Con	Uninf Baf	Inf Con	Inf Baf
Rapamycin	147	165	131	342
DMSO	189	239	193	178
PLGA	200	119	182	225
C-PLGA	182	125	121	166

Determination of uptake of DiO loaded PLGA and C-PLGA NPs using fluorescence microscopy and Flow Cytometry. To perform the qualitative and quantitative investigations on the uptake of DiO loaded PLGA and C-PLGA NPs, RAW 264.7 macrophages were grown to confluency. Thereafter, cells were seeded at a density of 100 000 cells/ml and allowed to attach overnight on a 12 well plate. Macrophages were then washed twice with PBS at pH 7.5, to remove any unattached cells. The attached cells were then subsequently treated with the DiO loaded C-PLGA NPs dissolved in D10 at a final NP concentration of 0.5 mg/mL, for 24 h. After the treatment period, the cells were washed three times with PBS to remove extracellular NPs. The cells were then fixed with 4% paraformaldehyde and incubated for 20 min at 25 °C, washed once with PBS, and stored at 4°C in the fridge in 1 ml PBS for 1 week. Thereafter, the plates were taken out of the fridge and thawed for 15 min. Thereafter, PBS was removed the plates and replaced with 500 µl DAPI working stock (0.1 -1ug/ml) in PBS. The plates were

then covered in aluminium foil and incubated for 10-15 min at room temperature. The plates were subsequently washed twice with PBS with agitation to remove access DAPI. The plates were stored for a maximum of 2 h, PBS was removed, and the plate was immediately imaged with an Eclipse Ti-U fluorescence microscope using the green laser channel.

Determination of uptake of DiO loaded PLGA and C-PLGA NPs Using Flow Cytometry. To investigate possible pathways of the PLGA and C-PLGA NP-uptake, RAW 264.7 macrophages were grown to confluency. Thereafter, the cells were seeded at a density of 100 000 cells/ml and allowed to attach overnight on a 12 well plate. Macrophages were then washed twice with PBS, to remove any unattached cells. To investigate whether the NPs were taken up via CME or CIE, the attached cells were independently pre-treated for 30 min with medium containing 10 µg/ml chlorpromazine and 5 µg/ml filipin, respectively [6, 7]. To investigate whether the uptake was via phagocytosis or micropinocytosis, the macrophages were pre-treated with 3 µg/ml of anti-Dectin-1 (Clec7a) monoclonal antibodies and 5µM of phenoxybenzamine and incubated at 37°C for 2 h and 1 h, respectively [8-10]. The pre-treated macrophages were then washed three times with PBS treated with the DiO loaded PLGA and C-PLGA NPs dissolved in D10 at a final NP concentration of 0.5 mg/mL, for 24 h. The cells were then washed with PBS and subsequently removed from the plate wells using trypsinEDTA. The lifted cells where then spun down at 10 000 x g for 5 min, the supernatant was discarded, and the pellets were reconstituted in fresh D10 media. Uptake was then quantified by measuring the fluorescence intensity of the DiO loaded PLGA NPs in untreated macrophages and in macrophages pre-treated with and without the uptake pathway inhibitors before exposure to the DiO loaded PLGA NPs. The mean fluorescence intensity was measured using a BD Accuri C6 Flow Cytometer (BD International, Germany) using 10000 events per sample, the fluorescence of DiO loaded NPs treated and non-treated RAW 264.7 macrophages was measured using the fluorescein isothiocyanate (FITC-A) channel. The fluorescence data was then analysed on the FlowJo v10.8.1 (BD Biosciences, Germany) software, first the scatter plot population was gated to remove the cell debris and noninternalized NPs. The remaining macrophages were then gated to remove doublets by changing the Y-axis from a side scatter-area (SSC-A) to forward scatter-width versus a forward scatter-area setting in the x-axis (FSC-W vs FSC-A), leaving only single cells. The single cells were then gated and measured for fluorescence while keeping at least 1% of the untreated macrophages in the gate using the FSC-W vs FITC-A settings to compensate for the autofluorescence of the macrophages. Triplicated independent experiments were conducted for each sample using 10 000 events per sample.

Bacteria strain for animal infection. *M. tuberculosis* H37Rv was obtained from the Trudeau mycobacteria culture collection (Trudeau Institute, Saranac Lake, New York). The bacterium was grown to log phase in 10% Oleic Acid/Albumin /Dextrose/Catalase (OADC) (Difco Detroit) enriched Difco Middlebrook 7H9 liquid medium containing 0.5% glycerol and incubated at 37°C for 21 days. The resulting *M. tuberculosis* inoculum was aliquoted into sterile and frozen at -80°C until further use. Bacterial viability was monitored by CFU assay. Bacterial colonies were counted after 21 days of plating (agar plates).

Physical observation and body weight monitoring. Mice were kept under close observation after each dosing (treatments administration on anaesthetized mice), with particular attention given to the physical signs of toxicity, including hypo/hyperactivity, convulsions, diarrhoea, tremors, lethargy, piloerection, salivation, straightening reflex, lacrimation, changes in skin and fur, hunched posture and arching. The scoring system was defined as follows: score zero for no discomfort or stress, score 1= for mild discomfort/stress, score 2= for moderate discomfort/stress, score 3= for severe discomfort/stress and finally, the

humane endpoint. Behavioural observations and systematic clinical evaluations were performed to ensure that mice found in a moribund condition or showing severe signs of distress and pain would be immediately subject to the humane endpoint. Mouse body weight was assessed three times per week before administration of anaesthesia/treatment for dose accuracy, and weight changes were calculated and recorded.

The weekly percentage changes were calculated as follows:

$$\text{Percentage change in body weight} = \frac{\text{End of week body weight - Initial body weight}}{\text{Initial body weight}} \times 100$$

Mice with a weight reduction of 20% or more were considered as the humane endpoint.

Quantification of cytokines and chemokines. Lung and spleen homogenates were centrifuged at 10 000 rpm for 10 min at 4°C and the supernatants were filtered using a sterile 0.22 µm syringe filters. A customized Milliplex Mouse Cytokine and Chemokine Magnetic Bead panel (cytokine/chemokine analytes with product ID MCYTOMAG-70k) was purchased from Merck (Merck Life Sciences, Germany). Cytokines IL-1β, IL-2, IL-4, IL-6, IL-10, TNF-α, IFNϒ, and chemokines CCL-2 (MCP-1), CCL-3 (MIP-1α), CCL-5 (RANTES) from supernatants were quantify by multiplex Luminex assay. Briefly, a 96-well plate was pre-wetted with 200 µL of wash buffer and mixed on a shaking plate for 10 minutes at room temperature (20-25°C) and decanted; 25 µL of each standard and control was added into the appropriate wells, and 25 µL assay buffer was added to background (0 pg/mL standard) and sample wells. Additionally, 25 µL of appropriate matrix solution was added to the background, standards and control wells followed by 25 µL of appropriate supernatant from mice homogenates being added to sample wells. Then, 25 µL bead mixture was added to each well and the plate was sealed, wrapped in foil and incubated overnight at 4 °C with shaking on a plate shaker. Next, the contents of the wells were removed and the plate was washed twice with 200 µL of wash buffer. Following that, 25 µL of detection antibody mixture was added into each well and the plate was sealed, covered with foil and incubated at room temperature with shaking on a plate shaker for 60 minutes. After that, 25 µL streptavidin-phycoerythrin was added to each well and the plate was sealed, covered with foil and incubated at room temperature with agitation on a shaker for 30 minutes. Thereafter, well contents were removed and the plate washed twice with 200 µL wash buffer, 150 µL Sheath Fluid PLUS was added to each well, the beads were resuspended on a plate shaker for 5 minutes and finally the plate was read on a Bio-Plex reader (Bio-Plex TM, Bio-Rad Laboratories, USA) to quantity the 12 analytes.

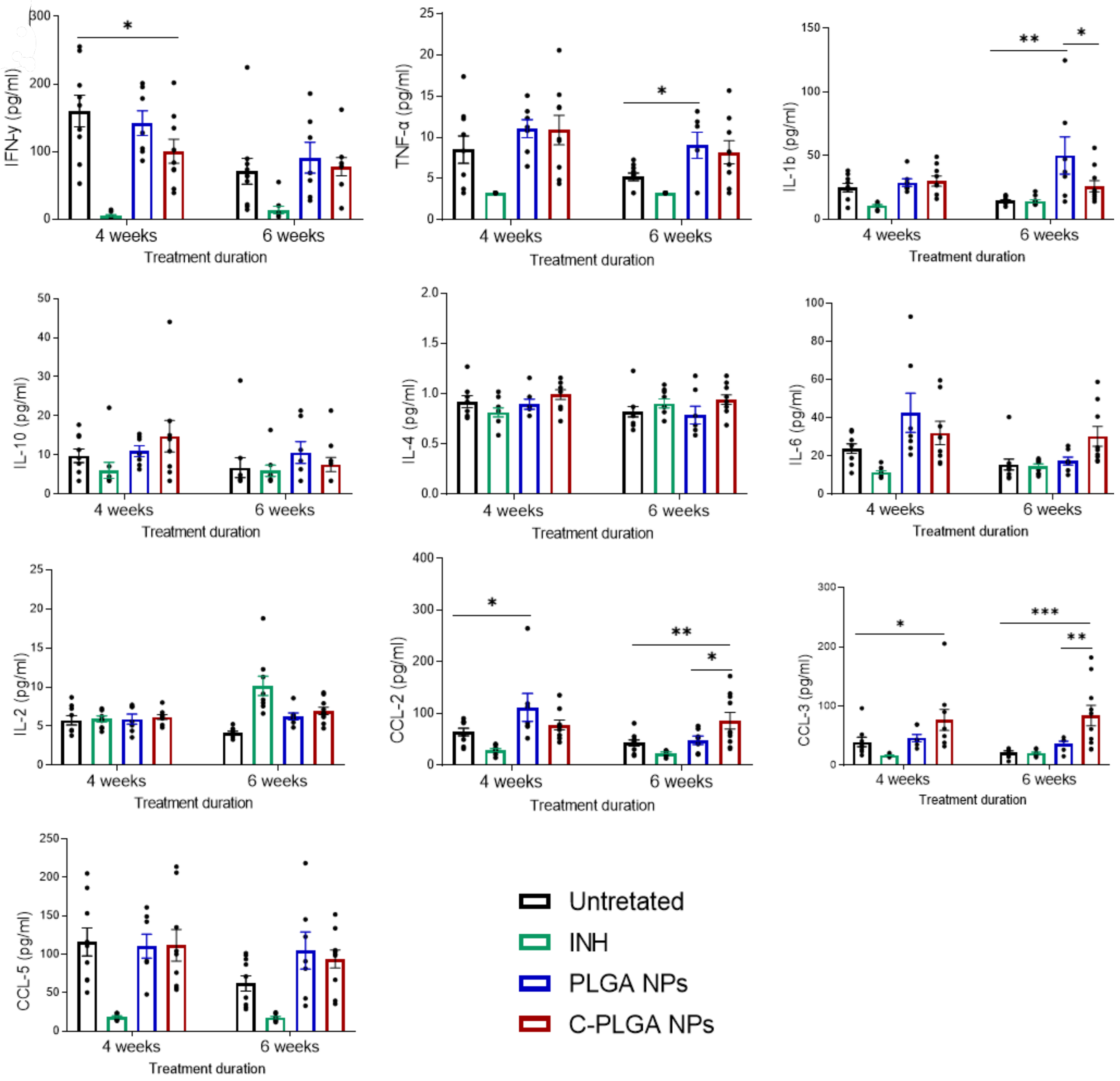

Figure S1: Cytokines and chemokines production from *M. tuberculosis* infected mice after 4 and 6 weeks of treatment with NPs. The pulmonary levels of IFN-γ, TNF-α, IL-1β, IL-10, IL-4, IL-6, IL-2, CCL-2, CCL-3 and CCL-5. Results are expressed as mean ± SEM; n= 7 -10 mice per group. The differences in means among groups were determined by ANOVA with a Tukey test or Dunnett's multiple comparisons test (*P< 0.05 ; **P< 0.01; ***P< 0.001, ****P <0.0001; P > 0.05= ns non-significant).

References

1. D'Souza, S., Du Plessis, S., Egieyeh, S., Bekale, R., Maphasa, R., Irabin, A., Sampson, S., and Dube, A.,2022 *Physicochemical and Biological Evaluation of Curdlan-Poly(Lactic-Co-Glycolic Acid) Nanoparticles as a Host-Directed Therapy Against Mycobacterium Tuberculosis.* J Pharm Sci. **111**(2): p. 469-478.

2. Leisching, G., Pietersen, R.-D., Van Heerden, C., Van Helden, P., Wiid, I., and Baker, B.,2017 *RNAseq reveals hypervirulence-specific host responses to M. tuberculosis infection.* Virulence. **8**(6): p. 848-858.

3. Leisching, G., Pietersen, R.-D., Mpongoshe, V., Van Heerden, C., Van Helden, P., Wiid, I., and Baker, B.,2016 *The Host Response to a Clinical MDR Mycobacterial Strain Cultured in a Detergent-Free Environment: A Global Transcriptomics Approach.* PLOS ONE. **11**(4): p. e0153079.

4. Ko, J.H., Yoon, S.-O., Lee, H.J., and Oh, J.Y.,2017 *Rapamycin regulates macrophage activation by inhibiting NLRP3 inflammasome-p38 MAPK-NFκB pathways in autophagy- and p62-dependent manners.* Oncotarget. **8**(25): p. 40817-40831.

5. Mejlvang, J., Olsvik, H., Svenning, S., Bruun, J.-A., Abudu, Y.P., Larsen, K.B., Brech, A., Hansen, T.E., Brenne, H., Hansen, T., Stenmark, H., and Johansen, T.,2018 *Starvation induces rapid degradation of selective autophagy receptors by endosomal microautophagy.* J Cell Biol. **217**(10): p. 3640-3655.

6. Bewersdorff, T., Vonnemann, J., Kanik, A., Haag, R., and Haase, A.,2017 *The influence of surface charge on serum protein interaction and cellular uptake: studies with dendritic polyglycerols and dendritic polyglycerol-coated gold nanoparticles.* Int J Nanomedicine. **Volume 12**: p. 2001-2019.

7. Rejman, J., Bragonzi, A., and Conese, M.,2005 *Role of clathrin- and caveolae-mediated endocytosis in gene transfer mediated by lipo- and polyplexes.* Mol Ther. **12**(3): p. 468-474.

8. De Quaglia E Silva, J.C., Della Coletta, A.M., Gardizani, T.P., Romagnoli, G.G., Kaneno, R., and Dias-Melicio, L.A.,2019 *Involvement of the Dectin-1 Receptor upon the Effector Mechanisms of Human Phagocytic Cells against Paracoccidioides brasiliensis.* J Immunol Res. **2019**: p. 1-11.

9. Elliott, J.A. and Winn, W.C.J.,1986 *Treatment of alveolar macrophages with cytochalasin D inhibits uptake and subsequent growth of Legionella pneumophila.* Infect Immun. **51**(1): p. 31-36.

10. Lin, H.P., Singla, B., Ghoshal, P., Faulkner, J.L., Cherian-Shaw, M., O'Connor, P.M., She, J.X., Belin De Chantemele, E.J., and Csányi, G.,2018 *Identification of novel macropinocytosis inhibitors using a rational screen of Food and Drug Administration-approved drugs.* Br J Pharmacol. **175**(18): p. 3640-3655.
